# Emission of Perfluoroalkyl Acids and Perfluoroalkyl Ether Carboxylic Acids to the Atmosphere from a Fluorochemical Industrial Park in China

**DOI:** 10.1021/acs.est.4c11394

**Published:** 2025-03-25

**Authors:** Bo Sha, Joost Dalmijn, Jana H. Johansson, Matthew E. Salter, Ian T. Cousins

**Affiliations:** † Department of Environmental Science, Stockholm University, Stockholm SE-10691, Sweden; ‡ Department of Thematic Studies - Environmental Change, Linköping University,, Linköping 581 83, Sweden; § Bolin Centre for Climate Research, Stockholm SE-10691, Sweden

**Keywords:** PFAS, air concentration, size-distribution, atmospheric transport, industrial emission

## Abstract

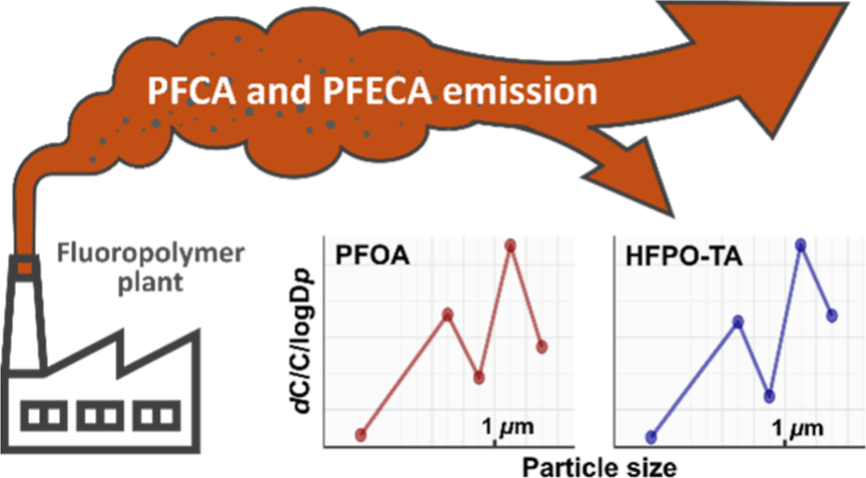

This study investigated the particle size distribution and atmospheric transport potential of perfluoroalkyl carboxylic acids (PFCAs) and certain perfluoroalkyl ether carboxylic acids (PFECAs) emitted from a mega fluoropolymer industrial park (FIP) in China. Ambient aerosols sampled in a residential area near the FIP were separated by a cascade impactor into five size fractions (<0.15 to 12.15 μm). Homologues of PFCAs (C5–C11) and five PFECAs were frequently detected in the samples (detection frequencies 40–100%), albeit not in all size fractions. Perfluorooctanoic acid (PFOA) exhibited the highest concentrations (6.5 to 2900 pg m^–3^). A noticeable mass mode in the >1 μm size range was observed for PFCAs and PFECAs in the samples that were directly influenced by wind from the direction of the FIP. Based on the PFOA concentrations in the aerosol samples, the emission rate of PFOA to air from the FIP was estimated to be 0.4–1.3 t year^–1^. Modeling results demonstrated that around 67% of the PFOA air emission was transported in the atmosphere above 1500 m in a 7 day continuous emission scenario, implying that the PFOA on <12.15 μm particles undergoes long-range atmospheric transport after being emitted from the FIP.

## Abstract 2

Size-resolved aerosol measurements revealed that direct emissions of PFASs on airborne particles from fluoropolymer production can travel several thousands of kilometers in air.

## Introduction

Perfluoroalkyl acids (PFAAs), which include perfluoroalkyl carboxylic acids (PFCAs) and perfluoroalkane sulfonic acids (PFSAs), are a subgroup of perfluoroalkyl and polyfluoroalkyl substances (PFASs). These substances have been extensively used in a variety of industrial and commercial applications since the 1950s.^1,2^ Salts of PFCAs, such as perfluorooctanoic acid (PFOA) and perfluorononanoic acid (PFNA), serve as processing aids for the emulsion polymerization manufacturing process of certain fluoropolymers,^3^ and these PFCAs are released into air and water during the production process.^4^ Emissions from fluoropolymer production facilities are estimated to account for the majority of the historical cumulative emissions of many PFCAs globally.^3,5,6^

Long-chain PFAAs, as defined by the Organisation for Economic Co-operation and Development (OECD), include PFCAs with ≥ 7 perfluoroalkyl carbons (C_*n*_F_2*n*+1_COOH, *n* ≥ 7) and PFSAs with ≥ 6 perfluoroalkyl carbons (C_*n*_F_2*n*+1_-SO_3_H, *n* ≥ 6).^7^ They are of global concern due to their ubiquitous presence and environmental persistence^8−12^ and their potential to bioaccumulate^13−15^ and cause health effects in biota and humans.^16,17^ A series of initiatives starting in 2000 with the aim of eliminating long-chain PFAAs prompted major global producers in Japan, Western Europe, and the United States to gradually reduce industrial emissions and substitute long-chain PFAAs and their precursors with short-chain homologues or other fluorinated substances such as perfluoroalkyl ether carboxylic acids (PFECAs).^3,18^ Conversely, PFAA production and use increased rapidly in Asia during the same period.^3,5^ For instance, China’s production of polytetrafluoroethylene (PTFE) increased from 0.66 × 10^4^ metric tons per year (hereafter t yr^–1^) in 1999 to approximately 6.4 × 10^4^ t yr^–1^ in 2012.^3^ It was estimated that between 2004 and 2012, industrial and domestic sources in China emitted 100–630 t of PFOA into the environment,^19^ contributing significantly to the global emissions of PFOA, which are estimated at 730–4773 t for the years 2003–2015.^3^ Elevated concentrations of PFCAs and PFECAs, including hexafluoropropylene oxide dimer acid (HFPO–DA) and hexafluoropropylene oxide trimer acid (HFPO–TA), have been reported in environmental media in areas directly impacted by fluoropolymer production plants in China.^20−25^ However, data on emissions from these industrial sources, particularly emissions into the air, remain scarce.

Atmospheric transport significantly contributes to the global presence of PFAAs, particularly in remote inland regions.^6,26^ It is estimated that about 16% of PFCA emissions from fluoropolymer production are released into the air,^3^ making this process a substantial source of PFCAs to the atmosphere. The majority of atmospheric PFAAs are thought to be associated with airborne particles,^27−29^ while studies have also shown that a substantial portion can be present in the gas phase.^23,30,31^ The atmospheric lifetime of these particles varies, lasting from weeks for fine particles (<2.5 μm) to minutes for coarse particles (>2.5 μm).^32^ Therefore, understanding the distribution of PFAAs on airborne particles of different sizes provides valuable insight into their atmospheric transport potential. Additionally, the size distribution of PFAAs on airborne particles can also affect their deposition in the alveolar region of the lung and therefore affect the exposure via inhalation^33^ While a few studies have investigated the mass-size distribution of PFAAs in urban^33−37^ or rural areas,^38^ measurements in regions directly impacted by industrial sources are limited. Previously, the mass-size distribution of PFOA was measured near the fence line of a fluoropolymer production facility in the United States.^39^ However, stacks are typically several tens of meters high. Given the lifetime of airborne particles, emissions from stacks can travel considerable distances before reaching ground level. Consequently, fence line measurements may not accurately represent direct emissions during fluoropolymer production.

In this study, we collected size-resolved aerosol samples in a residential area near a fluorochemical/fluoropolymer industrial park (FIP) using a 5-stage cascade impactor. We analyzed these samples for various PFAAs and PFECAs to assess their distribution in airborne particles of different sizes. Based on the size-distribution of PFAAs and PFECAs, we estimated the air emission rate of certain PFAAs from the FIP and evaluated their transport in the atmosphere using the HYSPLIT5 model. These results provide valuable information for assessing the impact of industrial sources on the transport of PFAAs and PFECAs in the atmosphere.

## Method

Aerosol sampling was conducted in a residential area in Zouping City, Shandong Province, China. A mega FIP is located in Huantai County of Zibo City, ∼30 km northeast of the sampling site, as shown in Figure S1 in the Supporting Information. Fluoropolymer products manufactured at the FIP include refrigerants, hexafluoropropylene (HFP, monomer, dimer, and trimer), PTFE, polyvinylidene fluoride (PVDF), fluorinated ethylene propylene (FEP), fluorine kautschuk material (FKM, a family of fluorocarbon-based fluoroelastomer materials commonly called as fluoro-rubber), etc. The fluoropolymer production capacities of the FIP were 4.65 × 10^4^ t of PTFE and 1 × 10^4^ t of PVDF in 2019.^40^ PTFE (and other fluoropolymer) production activities take place at multiple sites inside the FIP. The exhaust air is usually treated by a dry or wet electrostatic precipitator (DESP or WESP) before being released from 25–35 m stacks at each site.^41^ There were two PFOA production facilities in operation in the FIP until 2022, with a total production capacity of 30 t year^–1^. Exhaust air during PFOA production from both facilities was collected and incinerated, together with solid and liquid waste. The exhaust from the incineration was then emitted from a 60 m stack after a series of treatments (quench towers, wet scrubbers, alkaline washing towers, and WESP).^41^ Emission can also occur diffusively during the transport, storage, etc., of the raw materials and products. Elevated concentrations of PFOA, HFPO–DA, HFPO–TA, and other PFECAs measured in air, dust, and river samples in the surrounding area have been related to the emissions from the FIP.^20,24,25,42−46^ According to the records from the Ecology and Environment Bureaus (EEBs) of Zouping and Zibo cities,^47,48^ the FIP is the only fluorochemical and fluoropolymer facility in the region. The land between the FIP and our sampling site is predominantly agricultural. While other potential PFAS sources exist in the study area, including paper and textile manufacturing facilities, their contribution to atmospheric PFAA levels is likely minimal when air masses flow from the direction of FIP, which is the largest PTFE producer in Asia.

Sampling was carried out between March and April in 2019. A 5-stage impactor (Hauke) was used to collect aerosol samples. The cutoff sizes (*d*_50_) of the impactor stages were 0.15, 0.45, 1.35, 4.05, and 12.15 μm. Aluminum foil was used as a substrate on the impactor stages. Particles <0.15 μm were collected on a quartz fiber filter (QFF, Φ = 57 mm) placed after the impactor. Thus, each sample has 5 size fractions: <0.15 μm, 0.15–0.45 μm, 0.45–1.35 μm, 1.35–4.05 μm, and 4.05–12.15 μm. It should be noted that PFASs in the gas phase can sorb on the QFF during sampling,^28,31^ so the PFASs collected by the QFF included both particles phase and part of the gas phase. The impactor was placed on the flat roof of an apartment building (∼20 m above ground), and the inlet was ∼1.2 m above the roof. Ambient air was sampled daily at a flow rate of 150 L min^–1^ for ∼21 h from 11:00 to 08:00 the next day on 26 consecutive occasions (Table S1 in the Supporting Information). The samples were sealed in a 15 mL polypropylene tube after retrieval and stored at −18 °C before extraction.

The samples (aluminum foil and QFFs) were extracted in methanol and analyzed for PFASs on a Dionex UltiMate 3000 liquid chromatography system coupled to a Q-Exactive Plus HF Orbitrap mass spectrometer (LC-HRMS/MS; Thermo Scientific). Targeted analysis was performed for 8 PFCAs (C5–C12), 3 PFSAs (C4, C6 and C8), 17 PFECAs, and several other PFAS (Table S2 in the Supporting Information). The quantification is based on a calibration curve that includes mass-labeled internal standards (IS) and matching native standards of the targeted analytes. Technical grade PFOA (T-PFOA, 21% *br*-PFOA) and PFOS (T-PFOS, 32% *br*-PFOS) standards were used to quantify the branched and linear isomers. The recoveries of IS are summarized in Table S4 in the Supporting Information. The average IS-recoveries were generally between 30 and 80%. The mass-labeled PFPeA and HFPO–DA have relatively lower recoveries (∼20%) in the <0.15 μm and 0.15–0.45 μm size fractions. The reported concentrations were corrected for recoveries. Details regarding the extraction and instrumental analysis are provided in the Supporting Information.

The HYSPLIT model (version 5.2.0) was used to simulate the atmospheric transport of PFOA emitted from the FIP. The emission rate from the FIP in the year of 2019 was estimated by correlating observed concentrations with modeled concentrations at the sampling site (simulated at a constant emission rate of 1 pg h^–1^, details in the Supporting Information). This estimation was performed separately for each size fraction. Using the estimated size-resolved emission rate as an input, a second simulation to study the atmospheric transport of PFOA emitted from the FIP was conducted. The simulation used one-degree meteorological data from the Global Data Assimilation System (GDAS1) archive (https://www.ready.noaa.gov/archives.php). The emission period was set for 7 days (168 h), starting on March 30, 2019. The simulation covered a geographical area from 0° to 90°N latitude and 90°E to 90°W longitude, divided into 90 × 180 grid cells. It included 4 atmospheric layers: ground level (0 m, dry deposition by gravitational settling only), 0–1500 m above ground level (agl), 1500–5000 m agl, and 5000–10,000 m agl. The 24 h-average PFOA concentrations in each 1 ° × 1 ° grid cell at the end of the simulation period were then calculated. To account for uncertainties in the gridded meteorological data, an ensemble method, which is standard in the HYSPLIT model, was employed as in our previous study.^49^ This approach offsets the meteorological grid by a single grid point in latitude, longitude, and altitude, producing 27 concentration contour maps for all possible horizontal and vertical offsets. The arithmetic mean of these 27 maps was used to evaluate the atmospheric transport of PFOA.

## Results and Discussion

### PFAS Concentrations in the Aerosol Samples

**Figure 1:**
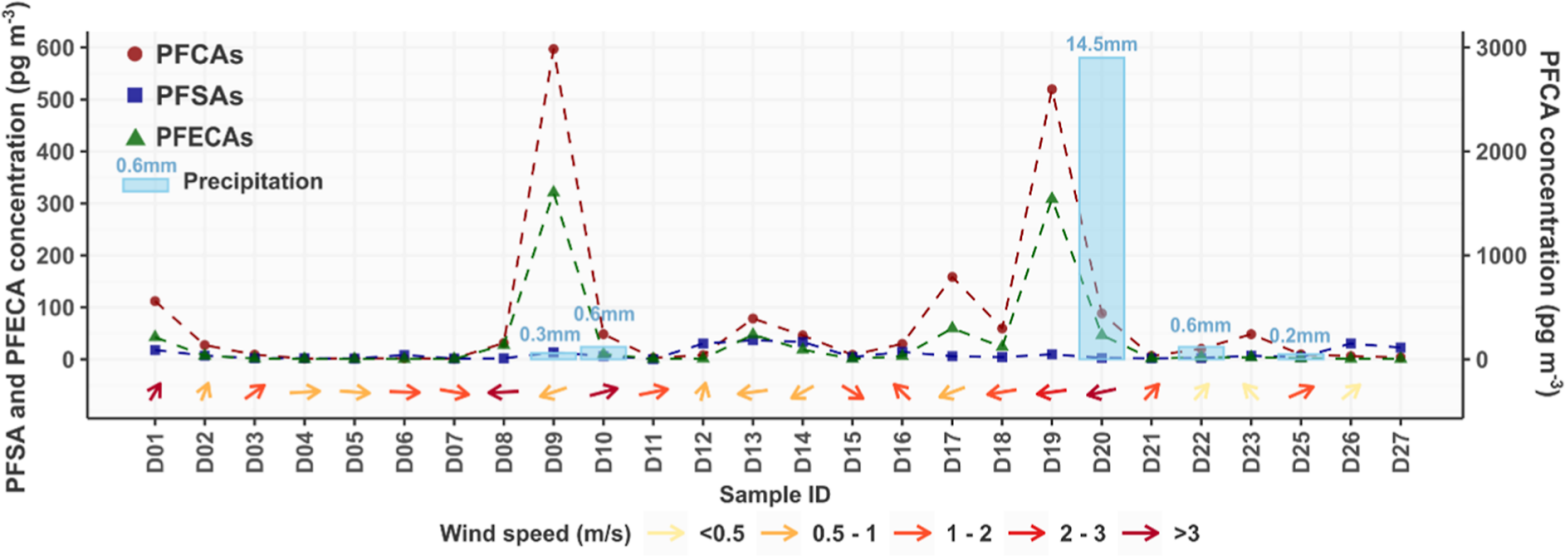
Concentrations of ∑PFCAs, ∑PFECAs, and ∑PFSAs in the samples (sum of the five size fractions) and the meteorological conditions during the sampling period. The left *y*-axis is for ∑PFECAs and ∑PFSAs, and the right *y*-axis is for ∑PFCAs. The directions and colors of the arrows indicate average wind direction and wind speed, respectively, and the blue bars indicate the total amount of precipitation during each sampling period. The dashed lines are for visual guidance.

An overview of the concentrations of ∑PFCAs, ∑PFECAs and ∑PFSAs in the samples and the meteorological conditions (wind direction, wind speed, and precipitation) during the sampling period is presented in Figure 1. Details on the detection frequencies and the concentration ranges of the targeted PFASs are presented in Figure S2 and Table S5 in the Supporting Information.

PFOA, including both the linear and branched isomers, was above the method detection limits (MDLs) in all samples across all size fractions. The ratios between linear and branched PFOA were consistent in all samples and all size fractions, with branched PFOA comprising 18 ± 2% of total PFOA, suggesting the use of electrochemical fluorination (ECF) in the FIP.^1^ C5–C7 and C9 PFCAs were detected in ≥ 92% of the samples (i.e., detected in at least one of the five size fractions), and their detection frequencies in the individual size fractions were more than 50% (Table S5). Though C10–C12 PFCAs were detected in 70–80% of the samples, their detection frequencies in the individual size fractions were only between 20 and 70% (Table S5). PFOA concentrations (linear and branched PFOA combined) ranged from 6.5 to 2900 pg m^–3^ (median = 120 pg m^–3^). The concentrations of C5–C7 PFCAs were generally between 0.1 and 10 pg m^–3^, and the concentrations of C9–C12 PFCAs were ten times lower than that of C5–C7 PFCAs, between < MDL and 0.1 pg m^–3^.

PFSAs were found above the MDLs in >70% of the samples. The detection frequencies in individual size fractions were generally ≥ 50%, except for PFBS (15–69%). The concentrations of PFOS (branched and linear combined) were 0.3–36 pg m^–3^, while the concentrations of other PFSAs were all below 1 pg m^–3^.

For the other targeted PFASs, five out of 17 PFECAs were detected in the aerosol samples, with HFPO–TA being the most frequently detected (92%). Perfluoro-3,6-dioxaoctanoic acid (PFO2OA or EEA) was detected in ∼70% of the samples and perfluoro-3,6,9-trioxadecanoic acid (PFO3DA) and HFPO–DA in ∼40% of the samples. An isomer of PFPeOPA, specifically perfluoro-5-oxanonanoic acid (CAS no. 174767-06-7) was detected in approximately 70% of the samples analyzed (Figure S3). Among the PFECAs observed, this PFPeOPA isomer exhibited the highest concentrations, reaching 260 pg m^–3^. Notably, this particular PFPeOPA isomer has been previously reported in a study examining follicular fluid samples in the Chinese population. In that study, the isomer was found at considerably higher concentrations (<MDL—147 ng mL^–1^) compared to other PFECAs (<MDL—11.8 ng mL^–1^).^50^ The concentrations of other PFECAs were generally comparable to that of PFOS and higher than that of C9–C12 PFCAs, except for PFO3DA, which is detected at lower concentrations than the PFSAs (Figure S2). The main component of F-53B, 2-(6-chloro-dodecafluorohexyloxy)-tetrafluoroethanesulfonic acid (9Cl-PF3ONS), was detected in 46% of the samples, with concentrations ranging between < MDL—1.6 pg m^–3^.

The changes in ΣPFCA and ΣPEFCA concentrations during the sampling period followed a similar pattern, as shown in Figure 1. Higher concentrations of ΣPFCAs and ΣPFECAs were associated with wind from the east (Figure S4), in which direction the FIP is located relative to the sampling site. In addition, the east wind appeared to be a principal contributor to the elevated PFOA concentrations in all five size fractions at the sampling sites (Figure S5). Correlation analysis showed that the concentrations of the C5–C8 PFCAs and the PFECAs were significantly correlated (Pearson’s *r* > 0.6, *p* < 0.05, Figure S6), suggesting that the FIP is likely a source of both the C5–C8 PFCAs and the PFECAs. ΣPFSAs did not reveal a clear pattern with wind direction (Figure S4). Significant correlations with the concentrations of PFCAs and PFECAs were observed for PFHxS (Pearson’s *r* > 0.6, *p* < 0.05, Figure S6) but hardly for the other PFSAs, implying that PFBS and PFOS may have a different origin than the PFCAs and PFECAs at the sampling site.

**Figure 2:**
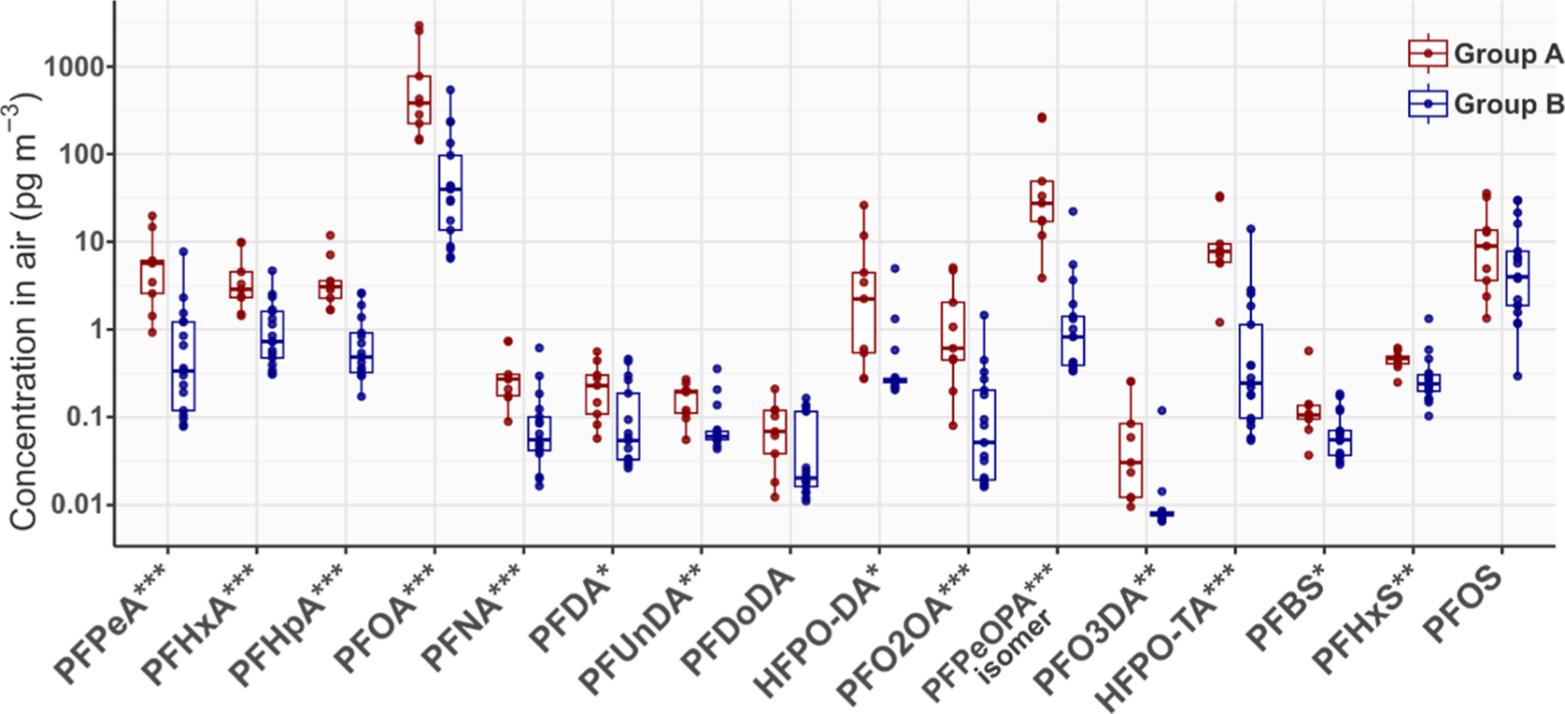
Boxplot of PFAA concentrations in the Group A (influenced by wind from the direction of the FIP) and Group B samples. The concentrations are for all size fractions combined. For values below the MDLs, 1/2MDLs are used in the plot. The horizontal lines inside the box represent the median values, and the lower and upper hinges correspond to the first and third quartiles (the 25th and 75th percentiles). The number of asterisks beside the compound names indicates whether there is a statistically significant difference (logarithm-transformed *t*-test) between the PFAS concentrations in the two groups at α = 0.05 (*), 0.01 (**), and 0.001 (***).

Based on the average of the wind direction and wind speed measured at the sampling site and FIP during each sampling period, the samples were categorized into two groups: Group A, influenced mostly by east winds (samples D08, D09, D13, D14, and D16–D20, Figure 1), and group B, comprising the rest of the samples. The concentrations of PFCAs in Group A samples were significantly higher than those in Group B (*t*-test, *p* < 0.05, Figure 2). The PFOA concentrations in Group A samples (140–3000 pg m^–3^, median = 380 pg m^–3^) were generally comparable to concentrations measured near the investigated FIP and other FIPs in China. Wang et al. reported PFOA concentrations of 42.8 to 9730 pg m^–3^ (median = 451 pg m^–3^) for 12 air samples collected at 12 sites within 10 km of the same FIP in November 2017.^25^ Dong et al. analyzed 30 air samples collected between July and October in 2022 at two sites within 5 km of the FIP, and the PFOA concentrations were 59.6–6650 pg m^–3^ (median = 887 pg m^–3^, gas phase and particle phase combined).^23^ Chen et al. deployed 8 passive air samplers for 44 days near two fluorochemical manufacturing facilities in Fuxin, China, and the PFOA concentration in these samples were 270–6400 pg m^–3^ (median = 690 pg m^–3^).^21^ Air samples collected in the city of Changshu, which is about 25 km away from another FIP, also showed comparable PFOA concentrations (mean = 560 pg m^–3^, maximum = 3515 pg m^–3^)^36^ to those in Group A samples. In contrast, PFOA concentrations in Group B samples (6.5–540 pg m^–3^, median = 39 pg m^–3^) were ∼10 times lower than those in Group A and were comparable to regions not directly influenced by the fluorochemical industry (generally <100 pg m^–3^), including several cities adjacent to the study area in the Bohai Rim.^35−37,51−55^ For comparison, the concentrations of PFOA in air samples from the Arctic are generally <5pg m^–3^.^49,56^

For the PFECAs, the concentrations in Group A samples were also significantly higher than those in Group B (*t*-test, *p* < 0.05, Figure 2). However, unlike PFOA, the combined concentrations of HFPO–DA and HFPO-TA in Group A (<MDL—35 pg m^–3^, median = 8 pg m^–3^) were 1–3 orders of magnitude lower than recent air measurements taken within 10 km of the FIP in 2022 (130–16,000 pg m^–3^, median = 3100 pg m^–3^).^23^ The ammonium salt of HFPO–TA is the main active ingredient of the processing aid used for PVDF production at the FIP.^20^ The sampling site in the present study is further away from the FIP (∼30 km) than the study in 2022 (<10 km), so lower HFPO–DA and HFPO–TA concentrations are expected due to the dilution during atmospheric transport over a longer distance. In addition, the production capacity of PVDF at the FIP has surged from <1 × 10^4^ t year^–1^ in 2019 to 2.5 × 10^4^ t year^–1^ in 2022,^41^ which may contribute to a greater use of HFPO-TA. Moreover, HFPO-TA concentrations may continue to rise as the FIP’s PVDF production capacity is expected to reach 5.5 × 10^4^ t year^–1^ in the near future.^57^

### Mass-Size Distribution of the Targeted PFAS

**Figure 3:**
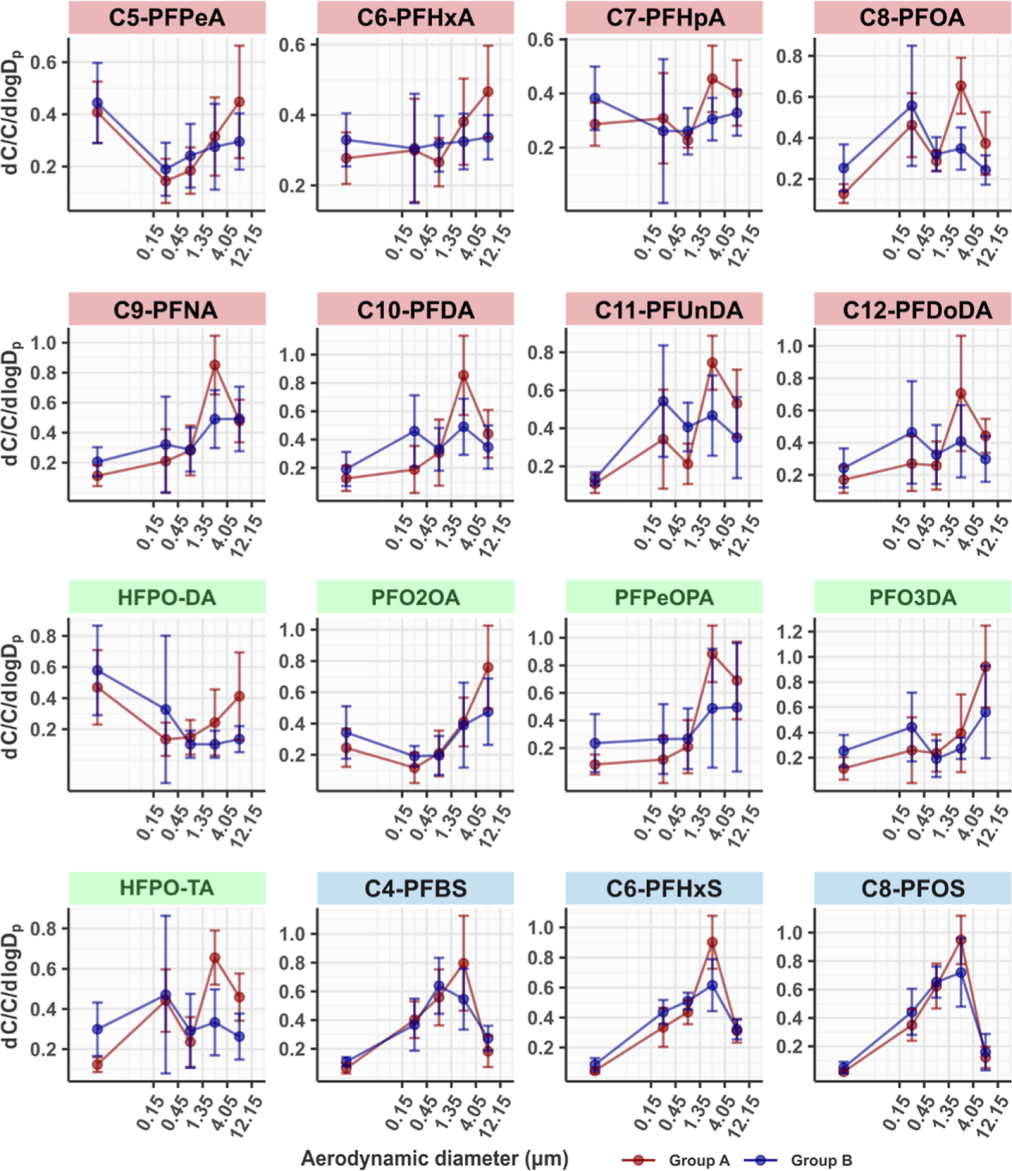
Mass-size distribution of PFCAs (shaded in red), PFCAs (shaded in green), and PFSAs (shaded in blue) in sample Group A and Group B. The distribution is normalized to the total concentration in each sample and averaged for each group. The lower limit of particle size on the QFFs was set to 0.001 μm. Error bars represent the standard deviations of *d*C/C/*d*logD_p_. For values below the MDLs, 1/2MDLs are used in the calculation.

The mass-size distributions of the targeted PFAS among the five size fractions in the two sample groups are shown in Figure 3. All PFCAs and PFECAs showed a noticeable mass mode in the >1.4 μm size range in Group A samples. For C5 and C6 PFCAs, HFPO–DA, PFO3DA, and PFO2OA, the supermicrometer mass mode was between 4.0 and 12 μm. For C7–C12 PFCAs, the PFPeOPA isomer and HFPO–TA, the main mass mode in Group A samples clearly appeared between 1.4 and 4.0 μm. However, this supermicrometer mass mode was much weaker and less obvious in Group B samples. Some compounds, such as C5–C7 PFCAs, HFPO–DA, and PFO2OA, have a considerable portion (30%–70%, Figure S6) present in the <0.15 μm size fraction in both sample Group A and Group B; however, it is difficult to conclude whether there was a mass mode in this size range. First, the QFF was supposed to collect all particles passed through the cascade impactor, so there is no definite cutoff size of the QFF. It was set to 0.001 μm for calculating the size distribution in Figure 3. Second, a substantial fraction of the PFAAs and PFECAs in the gas phase, which have been reported previously in air samples collected near the FIP (∼5 km),^23^ can sorb on the QFFs during sampling,^30,31^ resulting in overestimation of the concentrations in particles collected by the QFF. Further research with much higher particle size resolution in the <1 μm size range would help better elucidate the distribution of PFASs on the submicron particles.

PFOA and HFPO-TA, the two PFASs that are known to be used in the FIP, have similar mass-size distributions in both sample groups (Figure 3). PFOA and HFPO–TA revealed a bimodal distribution in Group A samples, with the main mass mode in the 1.4–4.0 μm range and a second mode in the 0.15–0.45 μm range. The percentages of the two compounds in the >1.4 μm size fractions in Group A samples were on average 49% for PFOA and 53% for HFPO–TA, which were significantly higher (*t*-test, *p* < 0.05) than those in Group B samples (on average 29% for both PFOA and HFPO–TA, Figure S7). On the contrary, the supermicrometer mass mode was less obvious in Group B samples and a much greater portion of PFOA (30% on average) and HFPO–TA (35% on average) was associated with the smallest size fraction in Group B than in Group A (on average 15% for PFOA and 14% for HFPO–TA, *t*-test, *p* < 0.001, Figure S7). In areas that are not directly impacted by emissions from industrial sources, the size distribution of PFOA showed a similar pattern to the Group B samples, which appeared to be dominated by lower aerosol size ranges. For example, PFOA was predominantly found (>60%) in the <0.14 μm size fraction of air samples collected in a semirural area in Germany.^38^ In Shanghai, China, PFOA revealed a main mass-mode in the 0.7–1.1 μm size range in aerosol samples collected during haze events.^33^

These differences in the size distribution between samples in Group A and Group B, also observed in other studies, imply that the PFCA and PFECA emission from the FIP may be more associated with coarse particles. The percentages of PFCAs and PFECAs in the >1.4 μm size fractions were significantly higher in Group A samples than in Group B samples (*t*-test, *p* < 0.05, Figure S7). Airborne particulate matter (PM) data obtained from nearby air monitoring stations indicated that PM_10_ concentrations increased during the sampling periods of Group A samples, while PM_2.5_ concentrations did not show a corresponding increase (Figure S8). This suggests that the shift of PFCAs and PFECAs’ distribution toward coarser particles is likely due to the increased concentration of PM between 2.5 and 10 μm (PM_2.5–10_) during the sampling of Group A. The total PM emissions from the stacks are regularly monitored by the FIP; however, PM_2.5_ and PM_10_ are not measured separately, making it uncertain whether the emissions from the FIP contribute to the elevated PM_2.5–10_ concentrations. Other sources, such as the coal-fired power plants in and around the FIP, can also affect the PM concentration at the sampling site. The resuspension of surface dust during dry windy weather can be a potential contributor to PFCAs and PFECAs in the coarse mode. However, as shown in Figure S4, the average wind speed during each sampling period was <4 m s^–1^, which may not be strong enough to cause impactful dust or soil suspension. Nonetheless, the increased percentage of PFCAs and PFECAs in the coarse size fractions in the Group A samples suggests that a large portion of these compounds emitted from FIP is likely associated with coarse particles.

It is reasonable to assume that a considerable fraction of PFOA is emitted with coarse particles. PFOA is a very surface-active substance, which favorably sorbs to surfaces. If PFOA was predominantly emitted as vapor and then sorbed on ambient particles, the main mass-mode in Group A would likely be in the <1 μm range, which is where the surface-area mode of airborne particles typically appears.^32^ However, considering that a bimodal size-distribution often implies discrete sources, our observations do not rule out emission in the gas phase. In a recent study near this FIP, researchers collected 30 air samples within a 5 km radius using glass fiber filters (GFFs) combined with PUF-XAD cartridges. Their analysis revealed that approximately one-quarter of both PFOA (23 ± 17%) and HFPO-TA (24 ± 15%) existed in the gas phase.^23^ The observed mass mode of particles <1 μm in Group A samples may partially result from gaseous PFASs being emitted and then sorbing on ambient particles, for example, via adsorption on the surface and partitioning to the water phase and/or organic matter (i.e., absorption).^58^ However, our ability to analyze gas-particle partitioning of PFAAs and PFECAs is limited by two factors: the low size resolution for particles <1 μm and the absence of data on both gaseous PFASs and the ambient particle number-size distribution.

Interestingly, in samples collected at ground level near the fence line of a fluoropolymer manufacturing facility, Barton et al.^39^ observed a bimodal PFOA mass-size distribution more similar to the Group B samples in our study. This difference between Barton et al.^39^ and our study is likely due to the differences in sampling location (e.g., distance) and emission conditions (e.g., stack height and exhaust treatment). Since particles take time to reach ground level after being emitted from the stack (depending on the stack height), fence line measurements may underestimate the impact of stack emissions.

The atmospheric transport of particles affects their size distribution through multiple mechanisms. Large particles settle out more quickly due to gravity as the distance from the source increases. Additionally, particle size changes with relative humidity as particles absorb moisture due to their hygroscopic properties. These changes in the water content and chemical properties (such as pH) influence how PFAAs and PFECAs partition between gas and particle phases. Furthermore, the FIP’s different treatment processes for exhaust gases from PTFE and PFOA production, along with potential emissions during product storage and transport, add complexity to the system. Understanding how these diverse factors collectively influence the PFOA size distribution from the FIP requires further investigation.

In contrast to PFCAs and PFECAs, C4, C6, and C8 PFSAs revealed a consistent unimodal mass-size distribution between the two sample groups, with the mass mode in the 1.4–4.0 μm size fraction. This is consistent with previous studies in a rural area in Germany (mass mode between 1.38 and 3.81 μm),^38^ in an urban area in Kyoto, Japan (between 2.2 and 4.9 μm),^59^ in Shanghai, China (between 2.1 and 5.8 μm),^33^ and other Asian cities (>2.5 μm).^35,36^ PFSA concentrations in the present study are also comparable to the observations in urban areas in China, which generally ranged from < MDL to ∼20 pg m^–3^.^23,25,33−37,51−55,60^ In addition, while correlations between PFSA and PFCA concentrations were observed in the size range 1.4–4.0 μm for all samples (Figure S6), further analysis showed that none of the PFSAs correlated with PFCAs in the Group A samples (*p* > 0.05). The comparable PFSA concentrations and size distribution observed in the present study and in urban areas in China suggest that the PFSAs at the sampling site share similar sources with those in other urban areas in China, rather than being contributed by the FIP. However, a new production facility for PFBS with a capacity of 40 t year^–1^, began operation in the FIP in 2024.^41^ Therefore, elevated PFBS concentrations are expected in the surrounding areas in the future.

### Estimated Emission to Air from the FIP and the Atmospheric Transport Potential

The estimation of PFOA emissions to air from the FIP was performed for each size fraction separately, as shown in Figure S9. Only samples in Group A were included in the estimation, which were directly influenced by the emission from the FIP. The *R*^2^ values of the weighted linear regression between the output from the HYSPLIT model and PFOA concentration in the samples were between 0.5 and 0.8 and the slopes were significantly greater than zero (*p* < 0.05). The upper and lower bonds of the emission estimate was calculated based on the 95% confidence interval (α = 0.05) of the slope of the linear regression. The estimated PFOA emission to air in 2019 was 0.8 t yr^–1^(0.4–1.3 t yr^–1^), with the five size fractions from <0.15 μm to 4.0–12 μm accounting for 8%, 27%, 13%, 29%, and 23% of the emission to air, respectively. Considering that the production capacity of PFOA at the FIP was 30 t yr^–1^ in 2019,^41^ approximately 1–4% of the PFOA produced and used in PTFE production was released into the air if the facility was running at its full capacity.

The emissions of C5–C7 PFCAs, HFPO–TA, PFO2OA, and the isomer of PFPeOPA were also estimated based on the linear relationship between the concentrations of PFOA and other PFAAs in the size-resolved aerosol samples after logarithmic transformation (Table S6). The *R*^2^ values of the log–log linear regressions were generally >0.8 except for PFO2OA in the 0.15–0.45 μm (*R*^2^ = 0.7) and 0.45–1.4 μm size fractions (*R*^2^ = 0.6). The results are presented as means (range within 95% confidence interval) in Table S7 in the Supporting Information. The estimated emissions of C5–C7 PFCAs to air in 2019 were 2 orders of magnitude lower than PFOA, with each between 2 and 29 kg yr^–1^, and were about 1–10% of their emission to water.^24^ The estimated HFPO-TA emission to air in 2019 was 2–40 kg yr^–1^, which is <1% of the estimated riverine discharge in 2015 (4.6 t yr^–1^).^24^ This is in line with the assumption that the majority of the PF(E)CA emission is to water. For comparison, on the basis of the fluoropolymer production capacity of the FIP in 2021, Feng et al. estimated that up to 1.89 t PFOA was emitted from the same FIP to the air.^40^ By assuming that HFPO oligomers (including HFPO–DA and HFPO–TA) have similar emission settings to PFOA, the authors also estimated that a maximum of 1.026 t HFPOs was emitted to air.^40^

It should be noted that the emission to air might be underestimated since emission from stacks lower than 20 m and from diffusive sources within the FIP, such as the storage and transport of the raw materials and products, were underrepresented. Particles >12 μm were also not collected by the cascade impactor. Feng et al. observed that PFAS concentrations in outdoor dust samples collected within 15 km from the FIP decreased exponentially with increasing distance,^40^ which suggests the impacts of larger particles (>10 μm) or low-stack emission. Additionally, previous studies have reported PFAAs and PFECAs in the gas phase in air samples collected near the FIP,^23^ but these were not considered in this study, which may lead to underestimation of the emission rates as well. Factors such as the relative humidity and temperature could also affect the size-distribution of particles during atmospheric transport,^61^ which can introduce uncertainty when estimating the size distribution at the emission source. Moreover, the meteorological file used in the estimation was converted from the observations at ground level meteorological stations near the sampling site and the FIP, which was likely an important source of uncertainty, since the meteorological data may not be representative enough for the whole study area.

**Figure 4:**
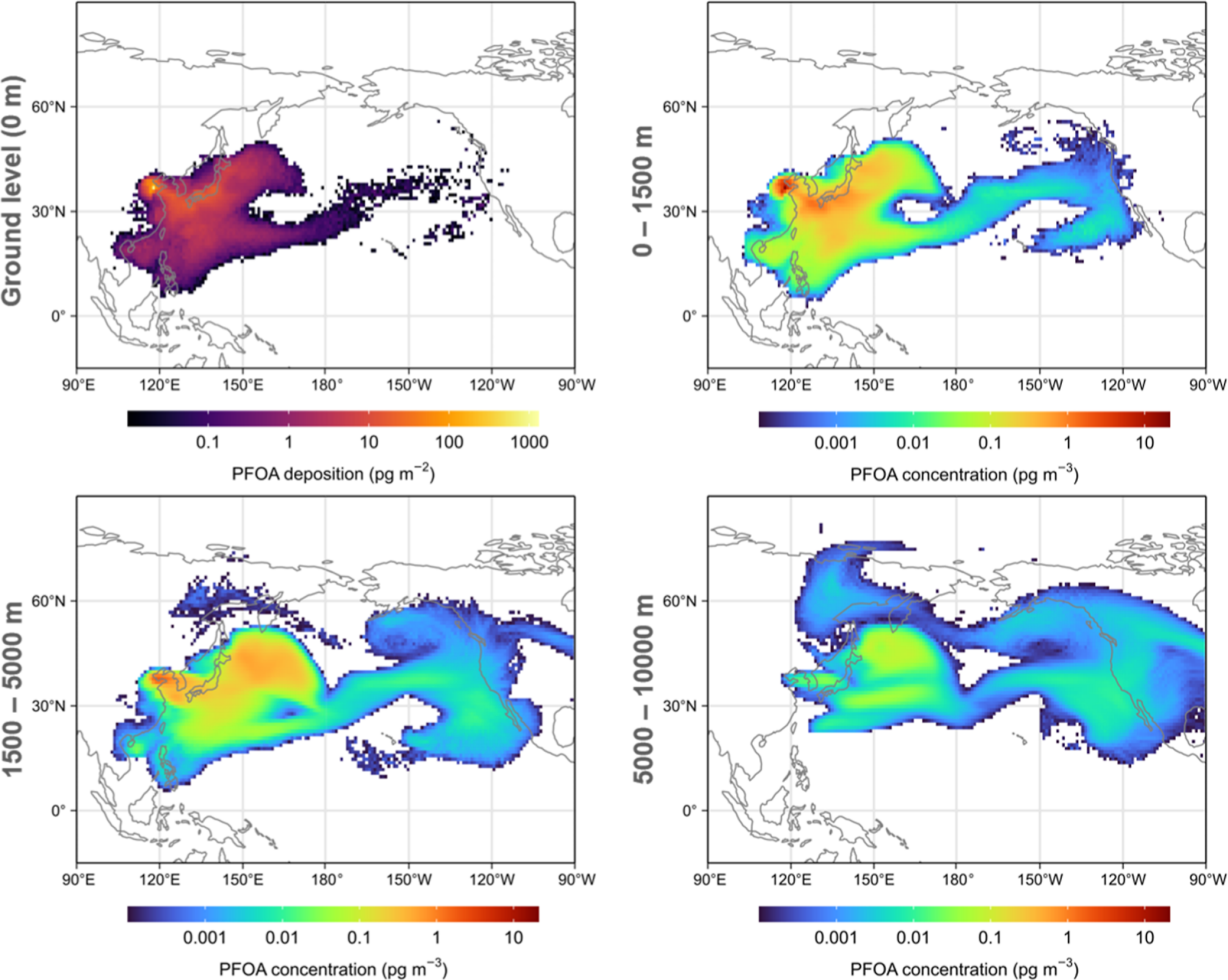
Total PFOA deposition (upper left) and 24 h average PFOA concentration at the end of the 7 days simulation by HYSPLIT5. The values in the grid cells are the arithmetic mean values of the ensemble simulation. Maps created based on the median values of the ensemble simulation are shown in Figure S11 in the Supporting Information.

Based on the estimated size-resolved emission rates, we investigated the atmospheric transport of PFOA emitted from the FIP using the HYSPLIT dispersion model. The concentration contour maps presented in Figure 4 indicate that PFOA emitted from the FIP is transported eastward in the atmosphere during the simulation period, potentially contributing to its concentration in the air over a large area of the northern Pacific. At the end of the 7-day simulation, only 0.4% of the total emission was deposited via gravitational settling. The deposition of PFOA was underestimated since gravitational settling was the only removal process that was considered in the simulation. Around 33% of the emission was in the lower atmosphere (<1500 m), while a considerable fraction reached altitudes >1500 m (57% between 1500–5000 m and 9.7% above 5000 m). This implies that, during the simulation period, PFOA emitted from the FIP can quickly enter higher atmospheric layers and potentially undergo long-range transport. It should be noted that the purpose of the simulation is to evaluate the atmospheric transport potential of PFOA emission from the FIP rather than modeling the PFOA deposition and air concentration on a regional scale, which requires good knowledge of various sources such as other FIPs, atmospheric transformation of precursors, and secondary emission from seawater via sea spray.

To demonstrate the influence of PFOA size-distribution on the atmospheric transport, we produced two additional emission scenarios in which all PFOA was released on a single size fraction: (1) the PFOA_1 μm_ scenario assumed a particle size of 1 μm, which is close to the geometric mean diameter 0.8 μm of the 5 size fractions, and (2) the PFOA_10 μm_ scenario assumed a particle size of 10 μm, which is close to the cutoff size 12.15 μm of the impactor’s inlet. All other parameters, including the total emission rate, were the same as those in the base scenario (PFOA_5-sizes_). Figure S12 compares the simulation results of the three scenarios. As expected, the dry deposition of PFOA by gravitational settling was the most impacted by the PFOA size distribution. In the base scenario, the total dry deposition at the end of the 7-day simulation is on average 10 times lower than the PFOA_10 μm_ scenario but 10 times higher than the PFOA_1 μm_ scenario. Although the coarser particle size scenario likely underestimates the contribution of FIP to PFOA in the air, particularly at higher altitudes, the differences in PFOA air concentrations were minor between the three scenarios (Figure S12). This is likely because the simulated air concentrations represent the average concentration within a volume of 1 ° × 1 ° grid area multiplied by the height of each atmospheric layer. The spatial resolution of the three scenarios is not high enough to capture the vertical distribution of PFOA in the lower atmosphere. Thus, in regional or global scale simulation of the air emission from FIPs, the modeled concentrations of PFAAs in the air might be insensitive to the size-distribution of the sources due to the relatively low spatial resolution. Nonetheless, a better knowledge on the size distribution of PFAS emission from industrial sources would help reduce the uncertainty in the modeled dry deposition fluxes.

The production of fluoropolymers in China has increased rapidly since the 2000s. It has been estimated that the fluoropolymer plants in China account for ∼60% of the global production capacity of PTFE and PVDF in 2022, including both local companies and international companies (e.g., Solvay and Chemours), and it is expected to continue increasing in the near future.^62^ Following China’s initiatives to restrict the use of long-chain PFAAs in 2022, many local fluoropolymer producers stopped using PFOA and switched to alternatives (short-chain PFCAs or PFECAs). For example, the FIP investigated in the present study ceased their production of PFOA in 2022 and has modified the facility to produce perfluorohexanoic acid (PFHxA) starting in 2024.^41^ Consequently, direct emission of PFOA from fluoropolymer production plants in China will likely decrease with simultaneously increasing emissions of the alternatives. While these alternatives share similarities to PFOA in terms of their use in fluoropolymer production, the differences in their physicochemical properties can influence the emission and atmospheric transport of the alternatives, which requires further research in the future.

## Supporting Information Available

Additional experimental details, materials, and methods, including additional figures and tables (PDF)
