# Supplementary material for: Emission of Perfluoroalkyl Acids and Perfluoroalkyl Ether Carboxylic Acids to the Atmosphere from a Fluorochemical Industrial Park in China
Source: Environ Sci Technol. 2025 Mar 25;59(13):6719–28. doi: 10.1021/acs.est.4c11394 (PMC11984109; doi:10.1021/acs.est.4c11394)
Supplement: Supplementary file 1 — es4c11394_si_001.pdf [file es4c11394_si_001.pdf]

Supporting Information for

Emission of perfluoroalkyl acids and perfluoroalkyl ether carboxylic acids to the atmosphere from a fluorochemical industrial park in China

Authors: Bo Sha[1*], Joost Dalmijn[1], Jana H. Johansson[2], Matthew E. Salter[1,3], Ian T. Cousins[1]

Affiliations:

[1]Department of Environmental Science, Stockholm University, SE-10691 Stockholm, Sweden.

[2]Department of Thematic Studies - Environmental Change, Linköping University, 581 83 Linköping, Sweden.

[3]Bolin Centre for Climate Research; Stockholm, SE-10691 Stockholm, Sweden.

[*]Corresponding author. Email: bo.sha@aces.su.se

Table of content

1. Supplementary text

2. Figures S1 – S8

3. Tables S1 – S7

Instrumental analysis

The samples (aluminum foil and QFFs) were first spiked with mass-labeled internal standards (IS) and then extracted by sonicating three times in 6 mL methanol (MeOH) for 20 min. The extract was evaporated to dryness and reconstituted in 50% MeOH and 50% 4mM ammonium acetate in MilliQ water to a final volume of 300 µL. Recovery standards (RS) were added prior to instrumental analysis.

PFAS were analyzed on a Dionex UltiMate 3000 liquid chromatography system coupled to a Q-Exactive Plus HF Orbitrap mass spectrometer (LC-HRMS/MS; Thermo Scientific). Extracts (25 µL) were injected onto an Acquity URLC® BEH C18 column (1.7 µm, 2.1x50 mm, Waters®) maintained at 30°C. The mobile phase consisted of A) 10:90 acetonitrile:water with 2 mM ammonium acetate; and B) 99:1 acetonitrile:water with 2 mM ammonium acetate. The flow rate of the mobile phase was 0.4 mL min^{-1} and the mass spectrometer was operated in negative electrospray ionization mode. Gradient conditions were: 90% A (and 10% B) from 0−0.5 min, 20% A from 0.5−8 min, 0% A from 8.1−11 min, and then 90% A from 11.1−13 min. Any background contamination originating from the instrument and mobile phase was delayed by using a "PFC isolator column" from Waters "PFC kit" placed before the injector. Targeted analysis was performed for 8 PFCAs (C5 – C12), 3 PFSAs (C4, C6 and C8), 17 PFECAs and several other PFASs (**Table S2** in the SI). The quantification is based on a calibration curve that includes all the targeted analytes. Technical grade PFOA (T-PFOA, 21% *br*-PFOA) and PFOS (T-PFOS, 32% *br*-PFOS) standard were used to quantify the branched and linear PFOA and PFOS isomers, respectively.

Field blanks were produced every five days by connecting the impactor and the backup filter to the pump for 1 minute and then treating the substrates and QFF as real samples. The method detection limit (MDL) was defined as the mean concentration in the field blanks plus three times the standard deviation (Mean+3×SD) and as 3 times the signal-to-noise levels (S/N) if the analytes were not detected in the field blanks. The method quantification limit (MQL) was defined as the mean concentration in the field blanks plus ten times the standard deviation (Mean+10×SD) or as 10 times the signal-to-noise levels (S/N). The MDLs and recoveries of the IS in the samples are presented in **Table S3 and S4 in the SI.**

Estimation of PFAS emission rates from the FIP

The emission rates of PFOA from the FIP to the air were estimated using the HYSPLIT model (version 5.2.0). If it is assumed that: 1) the emission from the FIP is the main source of PFOA in the air at the sampling site, 2) the emission rate (pg h^{-1}) is constant, and 3) the meteorological conditions are stable, the relationship between the emission rate and the observed concentration at the sampling site can be approximated by:

$$C_{PFOA} = D \times E_{PFOA} \quad (1)$$

Where C_{PFOA} is the measured concentration (pg m^{-3}) at the sampling site, D is the atmospheric dilution factor (h m^{-3}) and E_{PFOA} is the emission rate (pg h^{-1}). Assuming a constant emission rate of 1 g h^{-1} in HYSPLIT, the modeled PFOA concentration at the sampling site can be interpreted as the dilution factor:

$$C_{HYSPLIT} = D_{HYSPLIT} \times 1\ pg\ h^{-1} \quad (2)$$

The actual emission rate can then be estimated from the slope of the linear regression between the measured concentrations and modeled dilution factors:

$$C_{PFOA} = D_{HYSPLIT} \times E_{PFOA} \quad (3)$$

The simulation, with emissions originating from the FIP (36.97°N, 118.04°E, 50 m above ground) covered the same time period as the sampling campaign. The 24 h-average PFOA concentration

($C_{HYSPLIT}$, from 10:00 to 10:00 the next day) was calculated for the sampling site (33.86°N, 117.75°E). The PFOA emission was assumed to be associated only with the particulate phase, and the five size fractions were modeled separately. The geometric mean diameter of each size fraction was used in the simulations. There are two ground level meteorological monitoring stations in the study area. One is in Zouping City and the other one in Huantai County. Hourly meteorological data is available for the two stations, including 2-minute average wind direction and windspeed etc. The data of the two stations was retrieved from the China Meteorological Data Service Center (http://data.cma.cn/). The average of the meteorological data from the two stations was converted into a spatially homogeneous custom meteorological file with a 10 km resolution, covering a 250 by 250 km domain. The washout of PFOA in the air via wet deposition during atmospheric transport was not included in the simulation.

The estimation of PFOA emissions to air from the FIP was performed for each size fraction separately as shown in **Figure S9**. Only samples in Group A were included in the estimation, which were directly influenced by the emission from the FIP. The measured PFOA concentration and the model output were logarithm transformed and significant correlations (p<0.05) were observed (**Figure S10**). A weighted linear regression was employed to reduce the impact of outliers with high values. The R^2 values of the weighted linear regression between the output from the HYSPLIT model and PFOA concentration in the samples in **Figure S9** were between 0.5 – 0.8 and the slopes were significantly greater than zero (p<0.05).

The emissions of C5 – C7 PFCAs, HFPO-TA, PFO2OA and the isomer of PFPeOPA were also estimated based on the linear relationship between the concentrations of PFOA and other PF(E)CAs in the samples after logarithm transformation (**Table S6**). The R^2 values of the log-log linear regressions were generally >0.8 except for PFO2OA in the 0.15 – 0.45 μm (R^2 = 0.7) and 0.45 – 1.4 μm size fractions (R^2 = 0.6). The results are presented as means (range within 95% confidence interval) in **Table S7** in the SI.

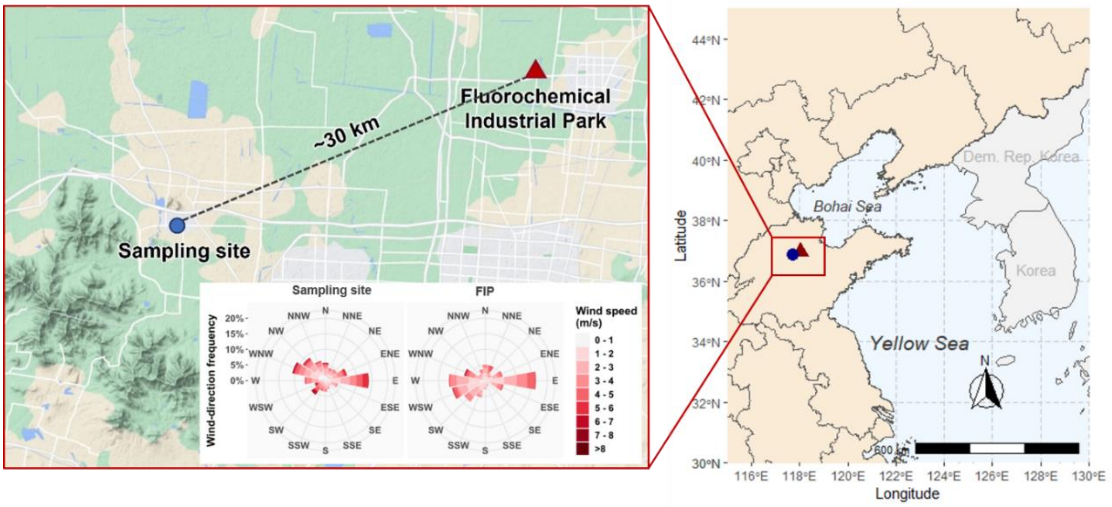

Figure S1. Locations of the fluorochemical industrial park and the sampling site. Hourly 2-minut average wind direction and wind speed data at ground level meteorological monitoring stations at the sampling site and the FIP was retrieved from the China Meteorological Data Service Center (http://data.cma.cn/).

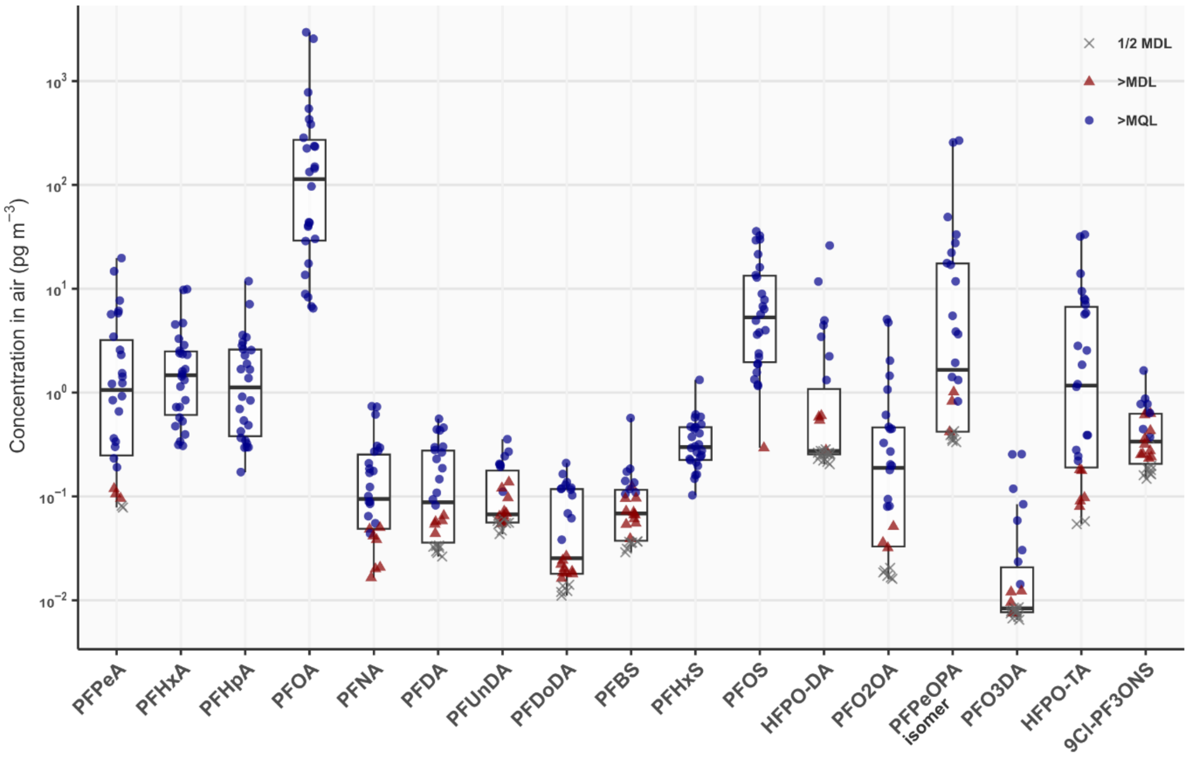

Figure S2. Boxplot of PFAA concentrations in the air samples (all size fractions combined). Red triangular and blue circles indicate that the concentration is above the MDL and above the MQL, respectively, in at least one size fraction of the sample. The grey crosses indicate that the concentrations in all size fractions are below the MDL and thus 1/2MDL is used in the plot. The horizontal lines inside the boxes represent the median values and the lower and upper hinges correspond to the first and third quartiles (the 25th and 75th percentiles).

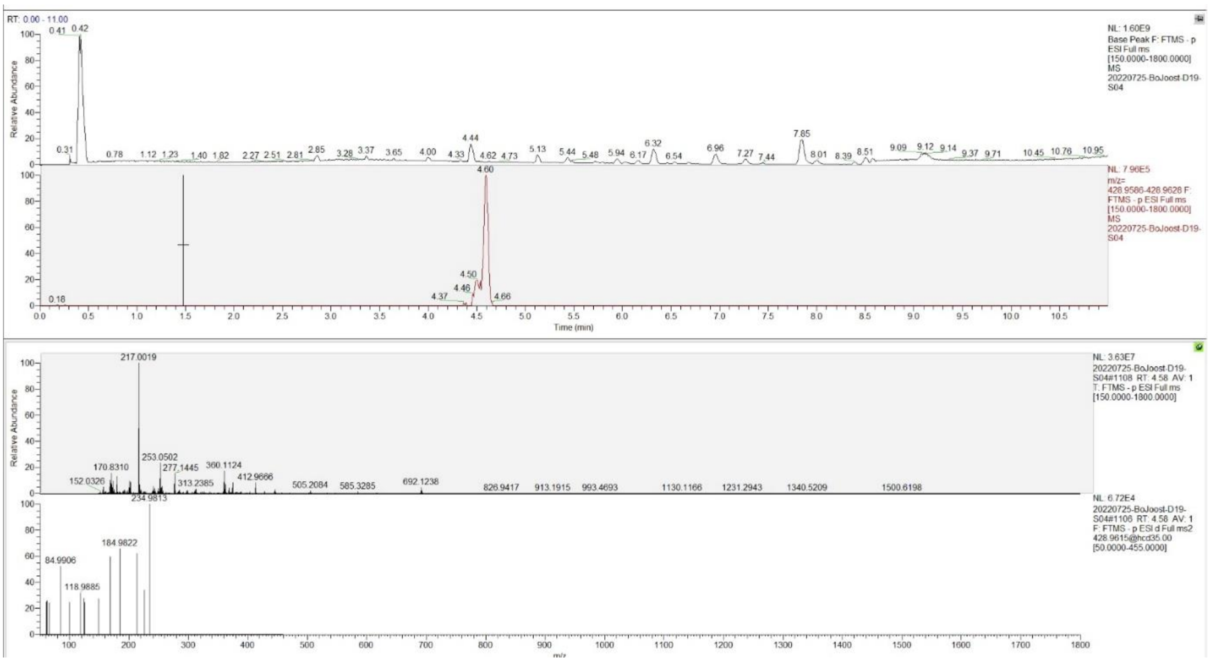

Figure S3. The MS2 spectra of the PFPeOPA isomer in one of the samples. The isomer is characterized by a daughter fragment of *m/z*=235 (C4F9O) instead of *m/z*=285 (C5F11O).

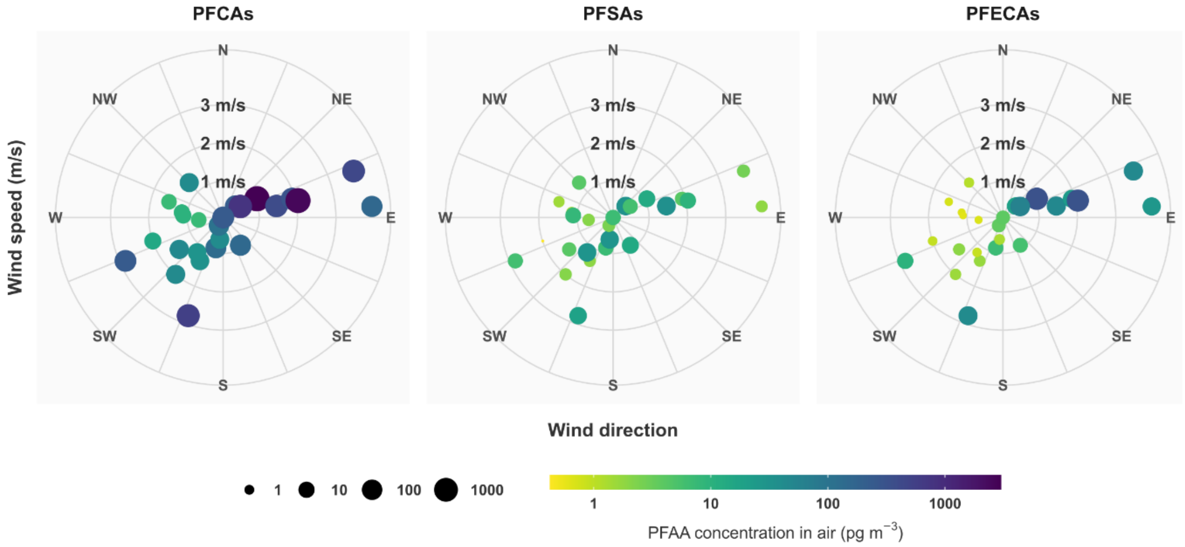

Figure S4. Plot of ∑PFCAs, ∑PFECAs and ∑PFSAs concentrations in the samples (sum of the five size fractions) against wind direction and wind speed on polar coordinates. The color and size of the markers indicate the concentration in each sample.

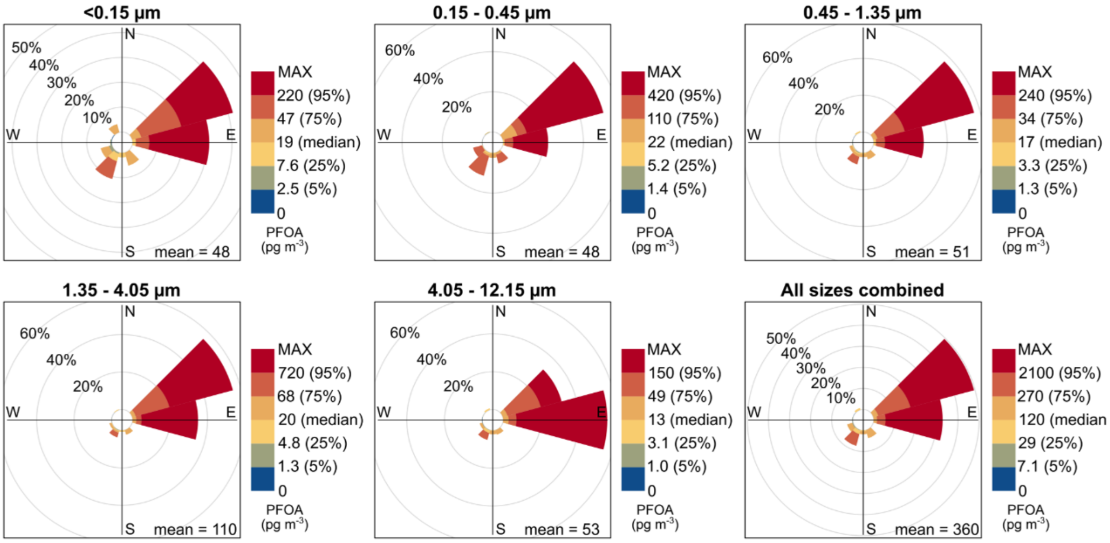

Figure S5. PFOA rose plot of the air samples. This figure plots PFOA concentration against wind direction. The rings indicate the contribution of samples influenced by certain wind direction to the observed mean concentration. The legend of each plot corresponds to the 5, 25, 50, 75 and 95 concentration percentiles in each size fraction. The plots were created using R with the openair package.

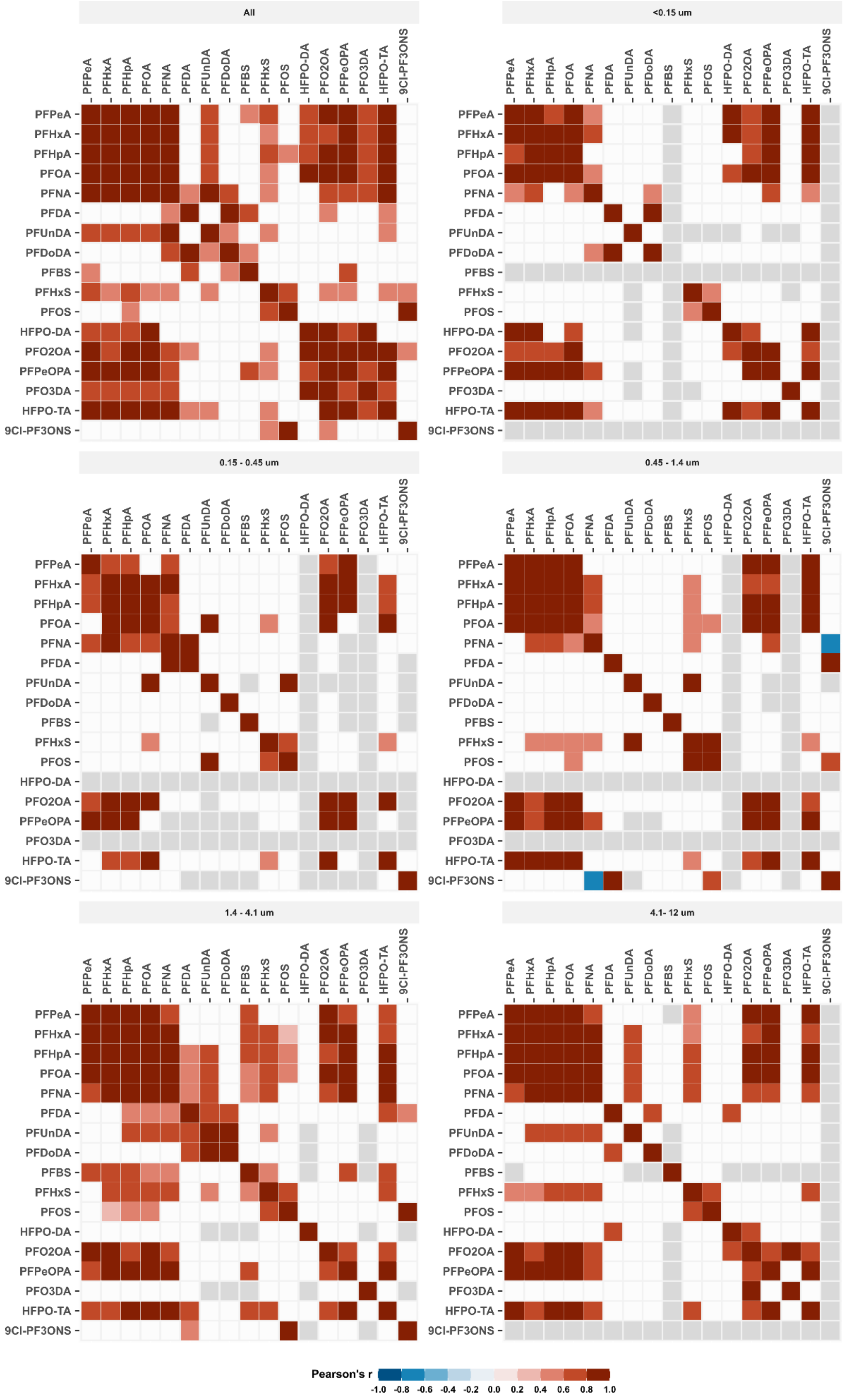

Figure S6. Correlations between each of the two target PFASs. Blank cells indicate insignificant correlation (*p*>0.05). Cells in grey indicate insufficient data for correlation analysis.

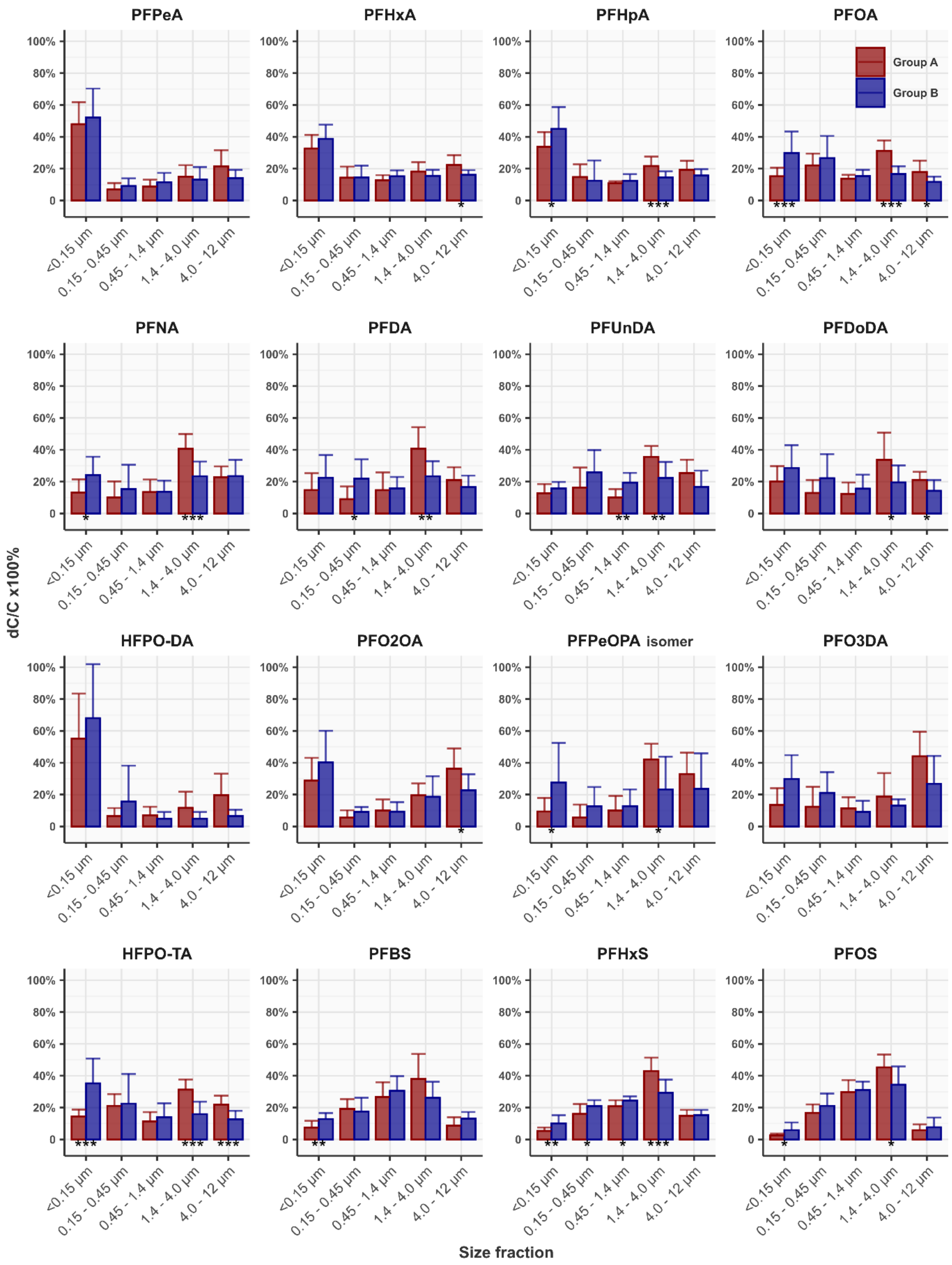

Figure S7. Percentage of PFAS concentrations in each size fraction. Error bars represent the standard deviations of the percentages. For values below the MDLs, 1/2MDLs are used in the calculation. The number of asterisks beside the compound name indicates whether there is significant difference (logarithm transformed t-test) between the percentages in the two groups at α = 0.05 (*), 0.01 (**) and 0.001 (***).

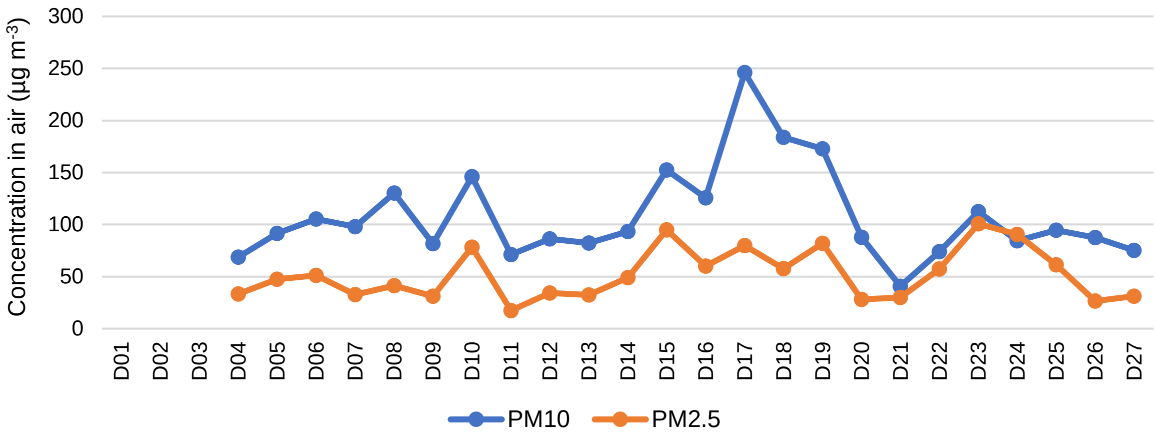

Figure S8. Average of PM2.5 and PM10 concentrations during the collection of each sample. Data obtained from monitoring stations close to the sampling sites.

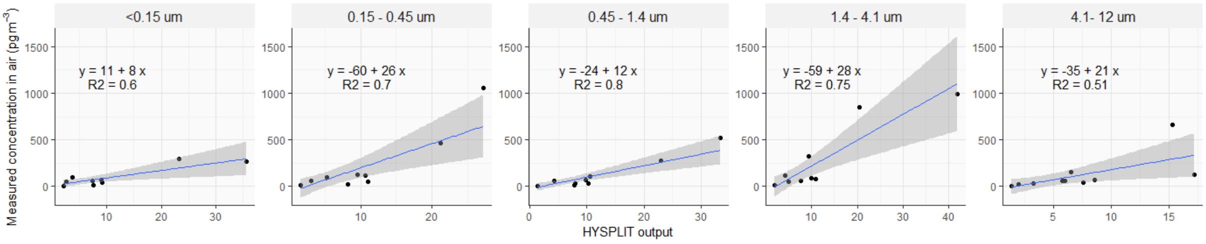

Figure S9. Weighted linear regression between the output of HYSPLIT model (x) and the measured PFOA concentration (y) in Group A samples. The shaded area indicates the 95% confidence (α=0.05) interval of the estimates.

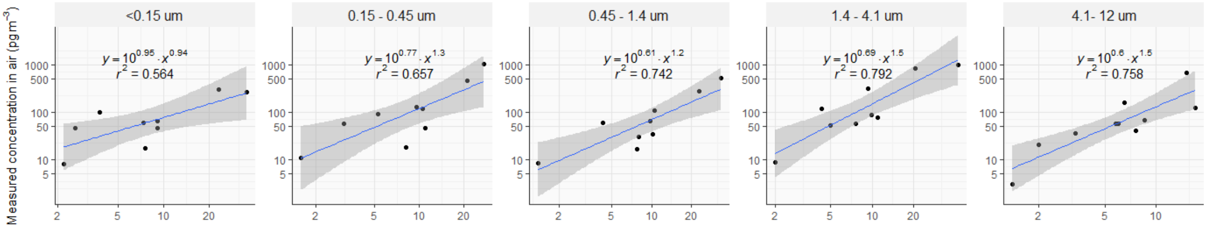

Figure S10. Log-log linear regression between the output of HYSPLIT model (x) and the measured PFOA concentration (y) in Group A samples. The shaded area indicates the 95% confidence (α=0.05) interval of the estimates.

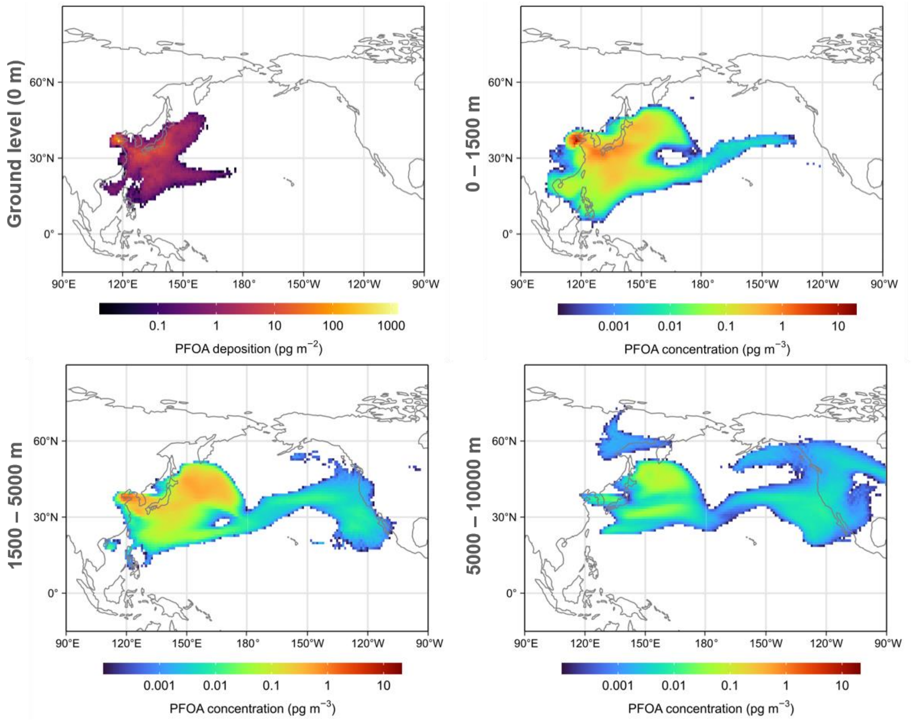

Figure S11. Total PFOA deposition (upper left) and 24-hours average PFOA concentration at the end of the 7-days simulation by HYSPLIT. The values in the grid cells are median values of the ensemble simulation.

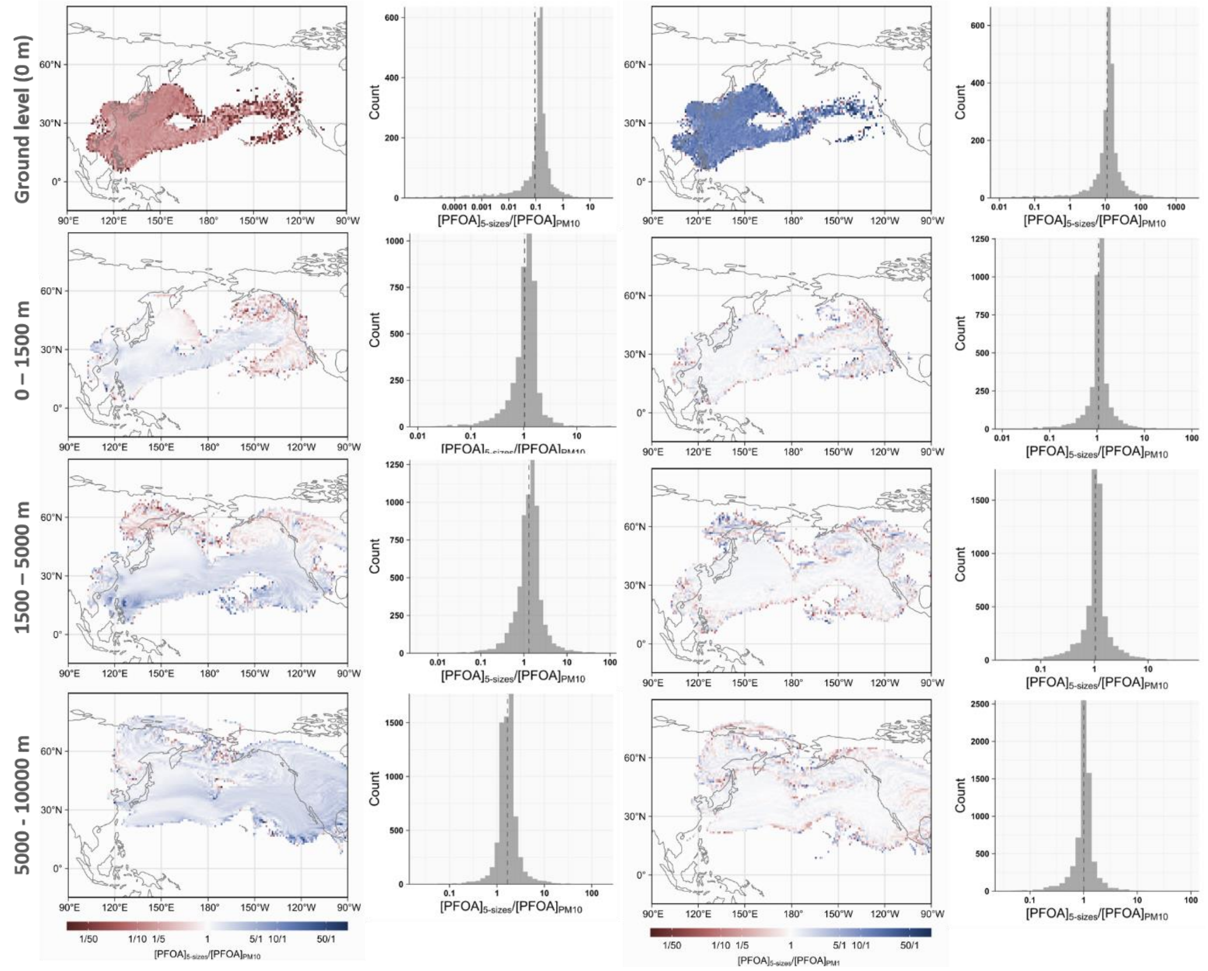

Figure S12. Ratios of simulated PFOA deposition and air concentration between the base scenario ($PFOA_{5\text{-sizes}}$) and the 10 µm ($PFOA_{10\mu m}$) and 1 µm ($PFOA_{1\mu m}$) scenarios. Blue color indicates higher values in the base scenario while red color indicates lower values in the base scenario. The histogram shows the distribution of the ratios in each map.

Table S1. Start time, end time and air volume of the samples

No.	ID	Start time (UTC)	End time (UTC)	Air volume (m^3)
1	D01	2019-03-20 11:45	2019-03-21 00:00	110.3
2	D02	2019-03-21 03:15	2019-03-22 00:00	186.8
3	D03	2019-03-22 02:50	2019-03-23 00:18	193.2
4	D04	2019-03-23 02:52	2019-03-24 00:08	191.4
5	D05	2019-03-24 02:40	2019-03-25 00:08	193.2
6	D06	2019-03-25 01:54	2019-03-25 23:54	198.0
7	D07	2019-03-26 03:25	2019-03-27 00:08	186.4
8	D08	2019-03-27 02:50	2019-03-28 00:20	193.5
9	D09	2019-03-28 03:07	2019-03-29 00:28	192.2
10	D10	2019-03-29 02:45	2019-03-30 00:23	194.7
11	D11	2019-03-30 02:57	2019-03-31 00:18	192.1
12	D12	2019-03-31 02:48	2019-04-01 00:08	192.0
13	D13	2019-04-01 02:27	2019-04-02 00:10	195.5
14	D14	2019-04-02 02:48	2019-04-03 00:17	193.4
15	D15	2019-04-03 02:48	2019-04-04 00:20	193.8
16	D16	2019-04-04 02:55	2019-04-05 00:18	192.5
17	D17	2019-04-05 02:45	2019-04-06 00:26	195.2
18	D18	2019-04-06 02:55	2019-04-07 00:11	191.4
19	D19	2019-04-07 02:35	2019-04-08 00:15	195.0
20	D20	2019-04-08 02:22	2019-04-09 00:02	195.0
21	D21	2019-04-09 03:52	2019-04-10 01:12	192.0
22	D22	2019-04-10 02:34	2019-04-11 00:23	196.4
23	D23	2019-04-11 02:52	2019-04-12 00:09	191.6
24	D25	2019-04-13 02:29	2019-04-14 00:10	195.2
25	D26	2019-04-14 02:28	2019-04-15 00:10	195.3
26	D27	2019-04-15 02:37	2019-04-16 00:19	195.3

Table S2. Native PFAS and the corresponding mass-labelled internal standard in the calibration curve.

Name	Formula	Internal standard	CAS	m/z used
**Perfluoroalkyl carboxylic acids (PFCAs)**				
Perfluoropentanoic acid (PFPeA)	$C_4HF_9O_2$	$^{13}C_5$ PFPeA	2706-90-3	262.9760
Perfluorohexanoic acid (PFHxA)	$C_6HF_{11}O_2$	$^{13}C_2$ PFHxA	307-24-4	312.9728
Perfluoroheptanoic acid (PFHpA)	$C_7HF_{13}O_2$	$^{13}C_4$ PFHpA	375-85-9	362.9696
Perfluorooctanoic acid (PFOA)	$C_8HF_{15}O_2$	$^{13}C_4$ PFOA	335-67-1	412.9666
Perfluorononanoic acid (PFNA)	$C_9HF_{17}O_2$	$^{13}C_5$ PFNA	375-95-1	462.9632
Perfluorodecanoic acid (PFDA)	$C_{10}HF_{19}O_2$	$^{13}C_5$ PFDA	335-76-2	512.9600
Perfluoroundecanoic acid (PFUnDA)	$C_{11}HF_{21}O_2$	$^{13}C_2$ PFUnDA	2058-94-8	562.9568
Perfluorododecanoic acid (PFDoDA)	$C_{12}HF_{23}O_2$	$^{13}C_2$ PFDoDA	307-55-1	612.9536
**Perfluoroalkyl sulfonic acids (PFSAs)**				
Perfluorobutane sulfonic acid (PFBS)	$C_4HF_9O_3S$	$^{18}O_2$ PFHxS	375-73-5	298.9429
Perfluorohexane sulfonic acid (PFHxS)	$C_6HF_{13}O_3S$	$^{18}O_2$ PFHxS	355-46-4	398.9366
Perfluorooctane sulfonic acid (PFOS)	$C_8HF_{17}O_3S$	$^{13}C_4$ PFOS	1763-23-1	498.9302
**Perfluoroalkylether carboxylic acids (PFECAs)**				
Perfluoro-3-methoxymethanoic acid (PMPA)	$C_4HF_7O_3$	$^{13}C_4$ PFBA	377-73-1	228.9738
Perfluoro-4-methoxybutanoic acid (PMBA)	$C_5HF_9O_3$	$^{13}C_5$ PFPeA	863090-89-5	278.9709
Perfluoro-3-tetrahydro-2-furancarboxylic acid (Furan-PFECA)	$C_5HF_7O_3$	$^{13}C_5$ PFPeA	65578-62-3	196.9836
Perfluoro-3,6-dioxaheptanoic acid (PFO2HpA)	$C_5HF_9O_5$	$^{13}C_2$ PFHxA	151772-58-6	200.9787
Hexfluoropropylene oxide dimer acid (HFPO-DA)	$C_6HF_{11}O_3$	$^{13}C_3$ HFPO-DA	13252-13-6	284.9780
Perfluoro-4-isopropoxybutanoic acid (PFIPBA)	$C_7HF_{13}O_3$	$^{13}C_4$ PFOA	801212-59-9	378.9642
Perfluoro-3,6-dioxaoctanoic acid (PFO2OA, EEA)	$C_6HF_{11}O_4$	$^{13}C_4$ PFHpA	80153-82-8	250.9760
3H-Perfluoro-3-[(3-methoxy-propoxy)propanoic acid (DONA)	$C_7H_2F_{12}O_3$	$^{13}C_4$ PFHpA	919005-14-4	376.9689
8-Iodoperfluoro(6-oxaoctanoic) acid (IPFOOA)	$C_7HF_{12}IO_3$	$^{13}C_4$ PFOA	948014-44-6	486.8701
Perfluoro-3,6-dioxadecanoic acid (PFO2DA)	$C_8HF_{15}O_4$	$^{13}C_4$ PFOA	137780-69-9	350.9691
2-Perfluoropentoxy-2,3,3,3-tetrafluoropropanoic acid (PFPeOPA)	$C_8HF_{15}O_3$	$^{13}C_4$ PFOA	504435-11-4	428.9608
Perfluoro-3,6,9-trioxadecanoic acid (PFO3DA)	$C_7HF_{13}O_5$	$^{13}C_4$ PFOA	151772-59-7	316.9674
Hexafluoropropylene oxide trimer acid (HFPO-TA)	$C_9HF_{17}O_4$	$^{13}C_5$ PFNA	13252-14-7	184.9834
Perfluoro-3,6,9-trioxatridecanoic acid (PFO3TriDA)	$C_{10}HF_{19}O_5$	$^{13}C_5$ PFDA	330562-41-9	466.9575
Hexafluoropropylene oxide tetramer acid (HFPO-TeA)	$C_{12}HF_{23}O_5$	$^{13}C_2$ PFDoDA	65294-16-8	350.9691
Hexafluoropropylene oxide pentamer acid (HFPO-PeA)	$C_{15}HF_{29}O_6$	$^{13}C_2$ PFDoDA	65150-95-0	516.9550
Hexafluoropropylene oxide hexamer acid (HFPO-HxA)	$C_{18}HF_{35}O_7$	$^{13}C_2$ PFDoDA	52481-85-3	682.9400

**Fluorotelomer sulfonic acids (FTSs)**				
4:2 fluorotelomer sulfonic acid (4:2 FTS)	$C_6H_5F_9O_3S$	$^{13}C_2$ 6:2 FTSA	75746-90-8	326.9743
6:2 fluorotelomer sulfonic acid (6:2 FTS)	$C_8H_5F_{13}O_3S$	$^{13}C_2$ 6:2 FTSA	27619-97-2	426.9679
8:2 fluorotelomer sulfonic acid (8:2 FTS)	$C_{10}H_5F_{17}O_3S$	$^{13}C_2$ 6:2 FTSA	251099-16-8	526.9615
**Fluoroalkanesulfonamides (FASAs)**				
Perfluorooctanesulfonamide (FOSA)	$C_8H_2F_{17}NO_2S$	$^{13}C_8$ FOSA	754-91-6	497.9462
N-ethylperfluorooctanesulfonamide (N-EtFOSA)	$C_{10}H_6F_{17}NO_2S$	d3-MeFOSAA	4151-50-2	525.9775
N-methylperfluorooctanesulfonamide (N-MeFOSA)	$C_9H_4F_{17}NO_2S$	d3-MeFOSAA	865-71-4	511.9618
Perfluorooctanesulfonamido acetic acid (FOSAA)	$C_{10}H_4F_{17}NO_4S$	d3-MeFOSAA	2806-24-8	555.9517
N-ethylperfluorooctanesulfonamido acetic acid (N-EtFOSAA	$C_{12}H_8F_{17}NO_4S$	d3-MeFOSAA	1336-61-4	583.9830
N-methylperfluorooctanesulfonamido acetic acid (N-MeFOSAA)	$C_{11}H_6F_{17}NO_4S$	d3-MeFOSAA	2355-31-9	569.9673
N-ethylperfluorooctanesulfonamidoethanol (N-EtFOSE)	$C_{12}H_{10}F_{17}NO_3S$	d3-MeFOSAA	1691-99-2	630.0248
N-methylperfluorooctanesulfonamidoethanol (N-MeFOSE)	$C_{11}H_8F_{17}NO_3S$	d3-MeFOSAA	24448-09-7	616.0092
**Chlorinated perfluoroalkylether sulfonic acids (Cl-PFESAs)**				
2-(6-chloro-dodecafluorohexyloxy)-tetrafluoroethane sulfonic acid (9Cl-PF3ONS)	$C_8HClF_{16}O_4S$	$^{13}C_4$ PFOS	756426-58-1	530.8956
11-Chloroeicosafluoro-3-oxaundecane-1-sulfonic acid (11Cl-PF3OUdS)	$C_{10}HClF_{20}O_4S$	$^{13}C_4$ PFOS	83329-89-9	630.8892

Table S3. Method detection limits (MDLs) and quantification limits (MQLs) of the target compounds that were detected in the samples. MDLs and MQLs for each sample were calculated by dividing the values in this table by the sample volume in Table S1.

**Compound**	**MDL (pg)**	**MQL (pg)**
PFPeA	5.2	14
PFHxA	9.6	15
PFHpA	3.0	6.0
PFOA-br	2.0	4.5
PFOA-l	14	25
PFNA	1.1	2.5
PFDA	2.1	5.6
PFUnDA	3.5	6.4
PFDoDA	0.9	2.4
PFBS	2.4	5.5
PFHxS	4.0	5.0
PFOS-br	4.6	12
PFOS-l	15	35
6:2 FTS	54	103
9Cl-PF3OUds	12	36
HFPO-DA	14	44
HFPO-TA	4.2	11
PFO3DA	0.5	1.1
PFPeOPA	26	78
PFO2OA	1.2	3.5

Table S4. Recovery of the mass-labeled internal standards (mean±sd).

**Size fraction**	**<0.15 µm**	**0.15 - 0.45 µm**	**0.45 - 1.35 µm**	**1.35 - 4.05 µm**	**4.06 - 12.15 µm**
MPFPeA	21±16%	22±13%	34±20%	46±19%	47±14%
MPFHxA	28±20%	31±19%	41±22%	56±19%	54±14%
MPFHpA	34±22%	37±20%	50±25%	67±22%	66±16%
MPFOA	38±21%	39±19%	53±22%	69±8%	72±6%
MPFNA	43±22%	44±21%	61±26%	80±23%	80±17%
MPFDA	31±12%	31±13%	38±15%	49±16%	47±13%
MPFUnDA	43±14%	41±16%	51±19%	65±20%	65±17%
MPFDoDA	48±11%	42±15%	50±19%	63±20%	62±19%
MPFHxS	72±5%	78±6%	77±6%	78±7%	79±7%
MPFOS	68±4%	73±4%	72±3%	73±4%	72±3%
M6:2 FTS	156±25%	137±37%	154±27%	165±27%	151±24%
MHFPO-DA	16±12%	20±12%	26±14%	36±17%	32±13%

Table S5. Summary of the detection frequencies and concentrations of the target compounds.

Compound	Detection frequency	Concentration (pg m^{-3})			Detection frequency in individual size fraction				
		Median	Min	Max	<0.15 µm	0.15 – 0.45 µm	0.45 – 1.4 µm	1.4 – 4.0 µm	4.0 – 12 µm
PFPeA	92%	1.1	<0.03	20	92%	58%	69%	65%	77%
PFHxA	100%	1.5	0.3	9.9	100%	85%	96%	96%	96%
PFHpA	100%	1.1	0.2	12	100%	73%	100%	100%	100%
PFOA-l	100%	99	5.7	2426	100%	100%	100%	100%	100%
PFOA-br	100%	16	0.8	526	100%	100%	100%	100%	100%
PFNA	100%	0.09	0.02	0.7	81%	46%	69%	92%	96%
PFDA	73%	0.09	<0.01	0.6	50%	38%	38%	69%	58%
PFUnDA	69%	0.07	<0.02	0.4	23%	27%	23%	54%	38%
PFDoDA	81%	0.03	<0.01	0.2	69%	35%	35%	54%	42%
PFBS	73%	0.07	<0.01	0.6	15%	46%	69%	65%	19%
PFHxS	100%	0.3	0.1	1.3	65%	100%	100%	100%	100%
PFOS-l	96%	3.7	<0.08	23	58%	96%	96%	96%	77%
PFOS-br	100%	1.6	0.1	12	69%	100%	100%	100%	77%
HFPO-DA	42%	<0.07	<0.07	26	35%	12%	12%	19%	31%
PFO2OA	77%	0.2	<0.01	5.1	73%	46%	50%	69%	69%
PFPeODA	73%	1.7	<0.13	267	50%	27%	42%	58%	54%
PFO3DA	46%	<0.01	<0.01	0.3	19%	15%	12%	19%	42%
HFPO-TA	92%	1.2	<0.02	33	92%	62%	77%	77%	69%
9Cl-PF3ONS	69%	0.3	<0.06	1.6	0%	31%	46%	65%	4%
6:2 FTS	19%	<0.27	<0.27	7.9	8%	12%	12%	8%	8%

Table S6. Log-log linear regression between the concentration of PFOA and other PFASs in the five size fractions ($\log_{10}$[PFAS] = $b + k \cdot \log_{10}$[PFOA]).

		**PFPeA**	**PFHxA**	**PFHpA**	**PFNA**	**HFPO-DA**	**PFO2OA**	**PFO2DA**	**PFPeOPA**	**HFPO-TA**
<0.15 µm	$k \pm CI_{95\%}$	1.2±0.2	0.6±0.1	0.6±0.1	0.5±0.2	1.3±0.6	1.3±0.2	0.8±1.5	1.4±0.2	1.2±0.2
	$b \pm CI_{95\%}$	-1.9±0.2	-1.1±0.1	-1.2±0.1	-2.4±0.2	-2.2±0.6	-3.1±0.2	-3.4±1.6	-2±0.2	-2.2±0.1
	R^2	0.8	0.80	0.83	0.42	0.74	0.85	0.30	0.95	0.90
0.15 – 0.45 µm	$k \pm CI_{95\%}$	0.6±0.4	0.6±0.1	0.9±0.1	0.7±0.3	n.a.	0.7±0.3	n.a.	1.7±0.6	1±0.2
	$b \pm CI_{95\%}$	-1.8±0.4	-1.5±0.1	-2.1±0.1	-2.7±0.3	n.a.	-2.6±0.3	n.a.	-3.3±0.7	-1.8±0.2
	R^2	0.5	0.80	0.84	0.67	n.a.	0.69	n.a.	0.87	0.83
0.45 – 1.4 µm	$k \pm CI_{95\%}$	0.9±0.1	0.5±0.1	0.6±0.1	0.5±0.2	n.a.	0.7±0.4	n.a.	1.1±0.4	1.1±0.2
	$b \pm CI_{95\%}$	-2±0.1	-1.3±0.1	-1.6±0.1	-2.5±0.1	n.a.	-2.4±0.3	n.a.	-1.5±0.3	-2.1±0.1
	R^2	0.9	0.82	0.91	0.58	n.a.	0.56	n.a.	0.79	0.85
1.4 – 4.0 µm	$k \pm CI_{95\%}$	0.7±0.1	0.5±0.1	0.6±0.1	0.6±0.1	0.4±1.1	0.8±0.2	0.8±0.5	1.2±0.2	1.1±0.1
	$b \pm CI_{95\%}$	-1.7±0.1	-1.3±0.1	-1.5±0	-2.2±0.1	-1.3±1.3	-2.6±0.1	-3.4±0.6	-1.6±0.2	-2.1±0.1
	R^2	0.9	0.87	0.94	0.77	0.15	0.82	0.73	0.93	0.92
4.0 – 12 µm	$k \pm CI_{95\%}$	1±0.1	0.6±0.1	0.6±0.1	0.5±0.1	0.6±0.6	1±0.2	0.8±0.6	1.3±0.2	1.2±0.1
	$b \pm CI_{95\%}$	-1.9±0.1	-1.2±0.1	-1.4±0.1	-2.1±0.1	-1.5±0.6	-2.5±0.1	-3.3±0.5	-1.5±0.1	-2.1±0.1
	R^2	0.9	0.85	0.91	0.76	0.42	0.85	0.50	0.96	0.94

n.a. – insufficient data for the linear regression

$CI_{95\%}$ - 95% confidence interval

Table S7 Estimated emission rate of certain PFCAs and PFECAs

		Emission rate in g h^{-1}				Emission rate in g h^{-1}	
	**PFOA**	Low	Median	High	**PFNA**	Low	Median	High
All		47	96	144		0.04	0.11	0.34
<0.15 µm		3	8	13		0.00	0.01	0.04
0.15 – 0.45 µm		13	26	38		0.00	0.02	0.15
0.45 – 1.4 µm		8	12	17		0.00	0.01	0.03
1.4 – 4.0 µm		16	28	39		0.02	0.04	0.09
4.0 – 12 µm		7	22	36		0.01	0.03	0.06
	**PFPeA**	Low	Median	High	**PFO2OA**	Low	Median	High
All		0.25	0.97	3.27		0.03	0.17	0.74
<0.15 µm		0.03	0.14	0.64		0.00	0.01	0.07
0.15 – 0.45 µm		0.01	0.11	1.22		0.00	0.02	0.20
0.45 – 1.4 µm		0.03	0.09	0.21		0.00	0.03	0.20
1.4 – 4.0 µm		0.07	0.21	0.64		0.01	0.04	0.12
4.0 – 12 µm		0.06	0.27	0.76		0.01	0.07	0.30
	**PFHxA**	Low	Median	High	**HFPO-TA**	Low	Median	High
All		0.58	1.41	3.20		0.23	1.12	4.60
<0.15 µm		0.11	0.28	0.63		0.02	0.08	0.29
0.15 – 0.45 µm		0.08	0.20	0.46		0.07	0.39	2.22
0.45 – 1.4 µm		0.11	0.19	0.32		0.04	0.11	0.34
1.4 – 4.0 µm		0.14	0.27	0.50		0.10	0.32	0.94
4.0 – 12 µm		0.13	0.35	0.79		0.05	0.32	1.13
	**PFHpA**	Low	Median	High	**PFPeOPA**	Low	Median	High
All		0.45	1.12	2.6		0.58	3.40	17.0
<0.15 µm		0.09	0.24	0.53		0.02	0.17	0.85
0.15 – 0.45 µm		0.04	0.12	0.39		0.002	0.13	8.8
0.45 – 1.4 µm		0.07	0.12	0.20		0.06	0.5	4.0
1.4 – 4.0 µm		0.12	0.23	0.39		0.31	1.5	6.4
4.0 – 12 µm		0.10	0.26	0.52		0.21	1.7	7.7
